# Smartly Assembled near‐Micron RF‐Responsive Agents Bridge Transarterial Embolization and Immunothermal Potentiation

**DOI:** 10.1002/advs.202600067

**Published:** 2026-03-26

**Authors:** Qitao Hu, Junkang Ding, Lingxiao Zhang, Zhou Tian, Cheng Zeng, Shaomin Zou, Wenjing Zhang, Tangye Zeng, Zhencang Gao, Zhouyi Sun, Gang Liu, Weiyu Chen, Zhe Tang

**Affiliations:** ^1^ Department of Surgery the Fourth Affiliated Hospital of School of Medicine and International School of Medicine International Institutes of Medicine Zhejiang University Yiwu China; ^2^ Interdisciplinary Nanoscience Center Aarhus University Aarhus Denmark; ^3^ State Key Laboratory of Molecular Vaccinology and Molecular Diagnostics National Institute of Diagnostics and Vaccine Development in Infectious Diseases Center for Molecular Imaging and Translational Medicine School of Public Health Xiamen University Xiamen China; ^4^ State Key Laboratory of Cellular Stress Biology Innovation Center for Cell Biology School of Life Sciences Xiamen University Xiamen China; ^5^ MOE Key Laboratory of Spectrochemical Analysis & Instrumentation College of Chemistry and Chemical Engineering Xiamen University Xiamen China; ^6^ Department of Respiratory and Critical Care Medicine Center for Oncology Medicine the Fourth Affiliated Hospital of School of Medicine and International School of Medicine International Institutes of Medicine Zhejiang University Yiwu China; ^7^ Zhejiang Key Laboratory of Precision Diagnosis and Treatment for Lung Cancer Yiwu China; ^8^ Department of Surgery The Second Affiliated Hospital Zhejiang University School of Medicine Hangzhou China

**Keywords:** combined immunotherapy, hepatocellular carcinoma, near‐micron nanomedicine, radiofrequency ablation, transarterial embolization

## Abstract

Hepatocellular carcinoma (HCC) remains a significant clinical challenge due to the limited efficacy of conventional treatments like surgery, radiofrequency ablation (RFA), and transarterial embolization (TAE). Here, we develop an innovative TAE and immunothermal therapy strategy based on smartly assembled near‐micron layered double hydroxide particles co‐loaded with STING agonist (NM‐LDHs‐cGP). By smartly responding to local charge fluctuations and microenvironmental changes, NM‐LDHs undergo self‐assembly and hierarchical stacking to form stable vascular embolic agents, thereby achieving superior embolization efficacy and excellent responsiveness to radiofrequency‐induced thermal stimulation. NM‐LDHs‐cGP activates the cGAS‐STING pathway, promotes dendritic cell maturation, and elicits robust CD8^+^ T and NK cell responses. Translational studies using human HCC tissues and immune cells confirm its ability to enhance RFA‐induced cytotoxicity and trigger potent innate and adaptive immune activation. These findings establish smartly assembled NM‐LDHs‐cGP as a promising paradigm for combinatorial embolization–thermal ablation–immunotherapy in liver cancer.

## 1 Main

Hepatocellular carcinoma (HCC) ranks as one of the most prevalent malignancies and the third leading cause of cancer‐related deaths worldwide [1, 2]. The treatment landscape encompasses various approaches, including liver resection, transplantation, thermal ablation therapies, and transarterial chemoembolization (TACE) [3, 4]. However, due to HCC's hypervascularity, insidious onset, aggressive nature, and rapid progression, recurrence remains common after treatment [5].

Radiofrequency ablation (RFA), which generates localized heat via high‐frequency alternating current, has been widely adopted [6]. Yet challenges such as large tumor size, poorly defined margins, and proximity to vessels often lead to incomplete RFA (iRFA) [7, 8]. Transarterial embolization (TAE)/TACE can mitigate the “heat sink” effect by reducing blood flow [9]. Despite their ability to disrupt the tumor's blood and nutrient supply, limitations persist, including incomplete vascular occlusion and rapid tumor recurrence following RFA [10, 11]. Additionally, TACE's chemotherapeutic effect is short‐lived, with repeated sessions often causing drug resistance and liver toxicity [12]. Conventional embolic agents merely form physical occlusions, lacking responsiveness to tumor environments or physical therapies like thermal ablation and radiation [13], which limits their precision and adaptability in combination therapies. Moreover, the general combination of TACE/TAE and RFA fails to activate systemic anti‐tumor immune responses efficiently [14, 15].

To date, various embolic agents have been explored, such as drug‐loaded microspheres [16], thermosensitive materials [17] and magnetic embolic microspheres [18], but many suffer from poor responsiveness or suboptimal embolizationy [19]. Layered double hydroxides (LDHs), a class of 2D, positively charged nanomaterials with the general formula [M_1−x_^2+^M_x_^3+^ (OH)_2_]^x+^ (A^n−^)_x/n_·mH_2_O, have shown promise in biomedical applications [20]. LDHs have been widely reported as delivery platforms capable of co‐encapsulating antigens, adjuvants, and small‐molecule drugs to elicit robust and broad‐spectrum immune responses [21]. Additionally, the metal components within LDHs suggest their potential for radiofrequency (RF) responsiveness, which enhances RFA efficacy through Joule [22]. Despite these advantages, previous studies have predominantly focused on nanoscale LDHs (about 100 nm), which are insufficient for effective embolization of tumor vasculature [23, 24]. The LDHs‐based agents with embolic functionality have yet to be developed.

Here, we present a multifunctional transarterial immunothermal strategy by synthesizing smartly assembled near‐micron‐scaled LDHs combined with cGAMP (NM‐LDHs‐cGP) to enhance TAE and RFA efficacy while activating antitumor immunity. Unlike nanoscale LDHs, our smartly assembled NM‐LDHs can smartly respond to local vascular charge and microenvironmental variations, transforming into larger size and exhibiting a markedly enhanced embolization capacity within tumor vasculature. They also exhibit RF‐responsive heating to boost RFA performance. Importantly, NM‐LDHs‐cGP adsorb tumor‐associated antigens and activate the cGAS‐STING pathway, promoting dendritic cell maturation and enhancing CD8^+^ T cell and NK cell cytotoxicity. These desirable features of NM‐LDHs‐cGAMP were further confirmed in clinical specimens, reinforcing their potential for clinical application. This study overcomes the limitations of conventional RFA and TAE/TACE therapies and introduces a novel combination treatment strategy with enhanced therapeutic efficacy.

## 2 Results and Discussion

### 2.1 Proteomic Landscape of Residual HCC Following RFA

**FIGURE 1:**
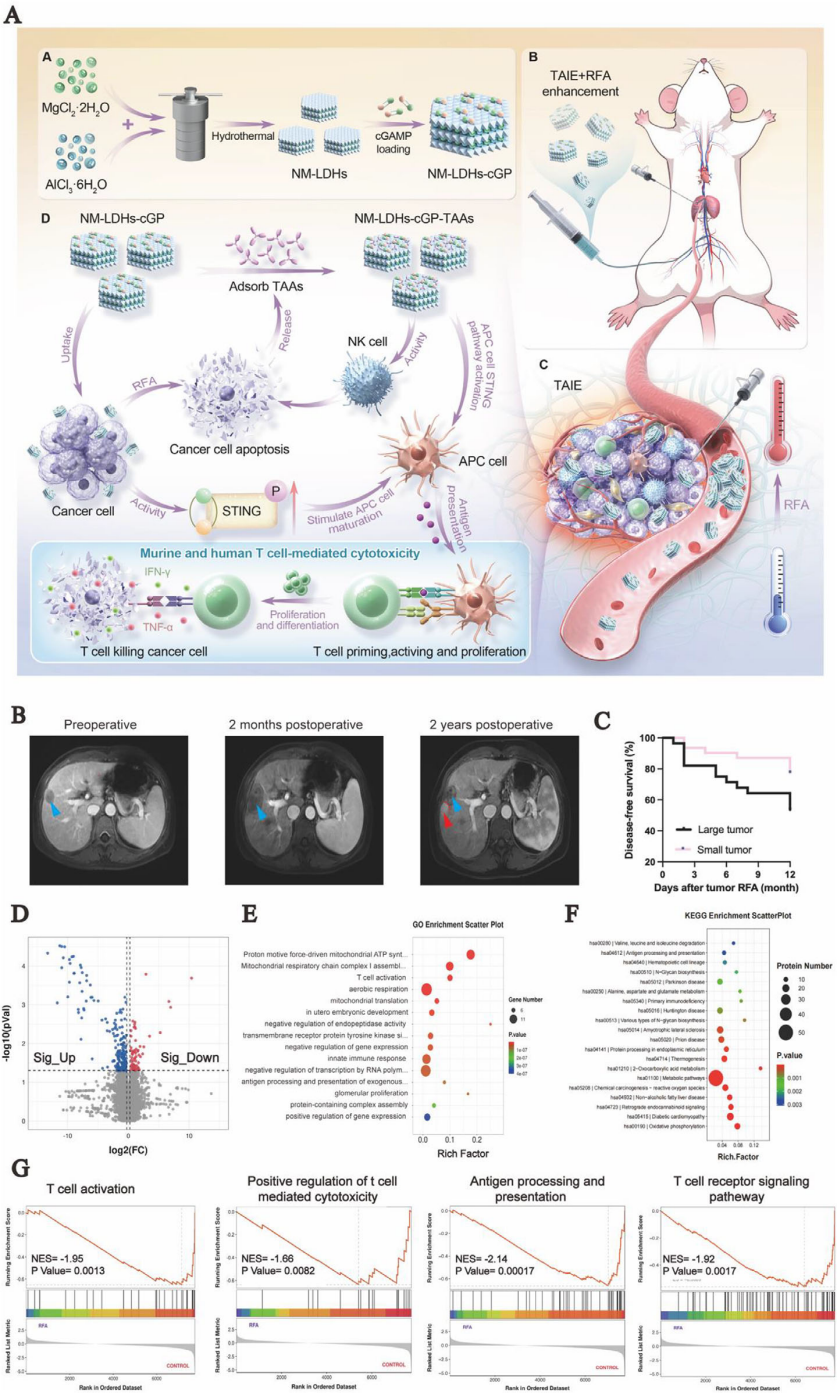
RFA‐Induced Immunosuppression and Tumor Recurrence in HCC. (B) Enhanced CT imaging results before and at different time points after RFA treatment. The blue arrow indicates the site of tumor radiofrequency ablation, while the red arrow denotes the location of tumor recurrence post‐ablation. (C) Kaplan‐Meier curve of disease‐free survival rate in patients with hepatocellular carcinoma after RFA. The black solid line represents small tumors, and the red dashed line represents large tumors. (D) Volcano plot of differentially expressed proteins in residual tumor tissues after RFA treatment compared to partial hepatectomy (*n* = 3). The horizontal axis represents log2(FC), and the vertical axis represents ‐log10(P‐value). Red dots indicate upregulated proteins, and blue dots indicate downregulated proteins. (E,F) GO, and KEGG enrichment analysis of the proteomics datasets. G, GSEA analysis of pathway activity after RFA treatment, including T cell activation, positive regulation of T cell activation, antigen processing and presentation and t cell receptor signaling pathway. The curves in each graph represent changes in the enrichment score (NES). P‐values and FDR values indicate statistical significance.

RFA is a critical curative treatment for HCC, but some patients still experience tumor recurrence after RFA (Figure 1B). Notably, our retrospective study shows that patients with larger tumors (tumor diameter ≥ 3 cm) have significantly lower disease‐free survival rates post‐RFA (Figure 1C; Table S1), likely due to incomplete RFA (iRFA) or immune alterations in the tumor microenvironment (TME).

To explore the impact of RFA on the TME, proteomic analysis was performed on tumor specimens from six HCC patients—three treated with RFA and three undergoing partial hepatectomy. Protein quantification reproducibility was confirmed by Pearson's correlation (Figure S1A). Compared to hepatectomy samples, 74 proteins were upregulated and 199 downregulated in residual tumor tissues near the ablation zone in the RFA group (Figure 1D). The top 50 differential proteins were shown in Figure S1B. Clustering and enrichment analyses revealed significant alterations in immune‐related pathways (Figure 1E,F; Figure S1C). Gene Ontology (GO) analysis revealed significant alterations in both innate and adaptive immune responses, as well as T cell activation (Figure 1E). Concurrently, Kyoto Encyclopedia of Genes and Genomes (KEGG) pathway analysis demonstrated a marked enrichment in antigen processing and presentation pathways (Figure 1F). Additionally, Gene Set Enrichment Analysis (GSEA) demonstrated reduced activity in T cell receptor signaling, MHC class protein complex binding, positive regulation of T cell activation and NK cell cytotoxicity after RFA (Figure 1G). These results suggest residual tumors adopt an immunosuppressive microenvironment post‐RFA, potentially driving recurrence.

Given HCC's hypervascularity, TAE can reduce tumor perfusion and improve RFA thermal efficiency. However, benefits remain limited due to incomplete tumor eradication and lack of added functionality. Developing embolic agents that combine TAE, RFA enhancement and additional antitumor effects remains a critical challenge for advancing HCC therapy.

### 2.2 Preparation and Characterization of NM‐LDHs

**FIGURE 2:**
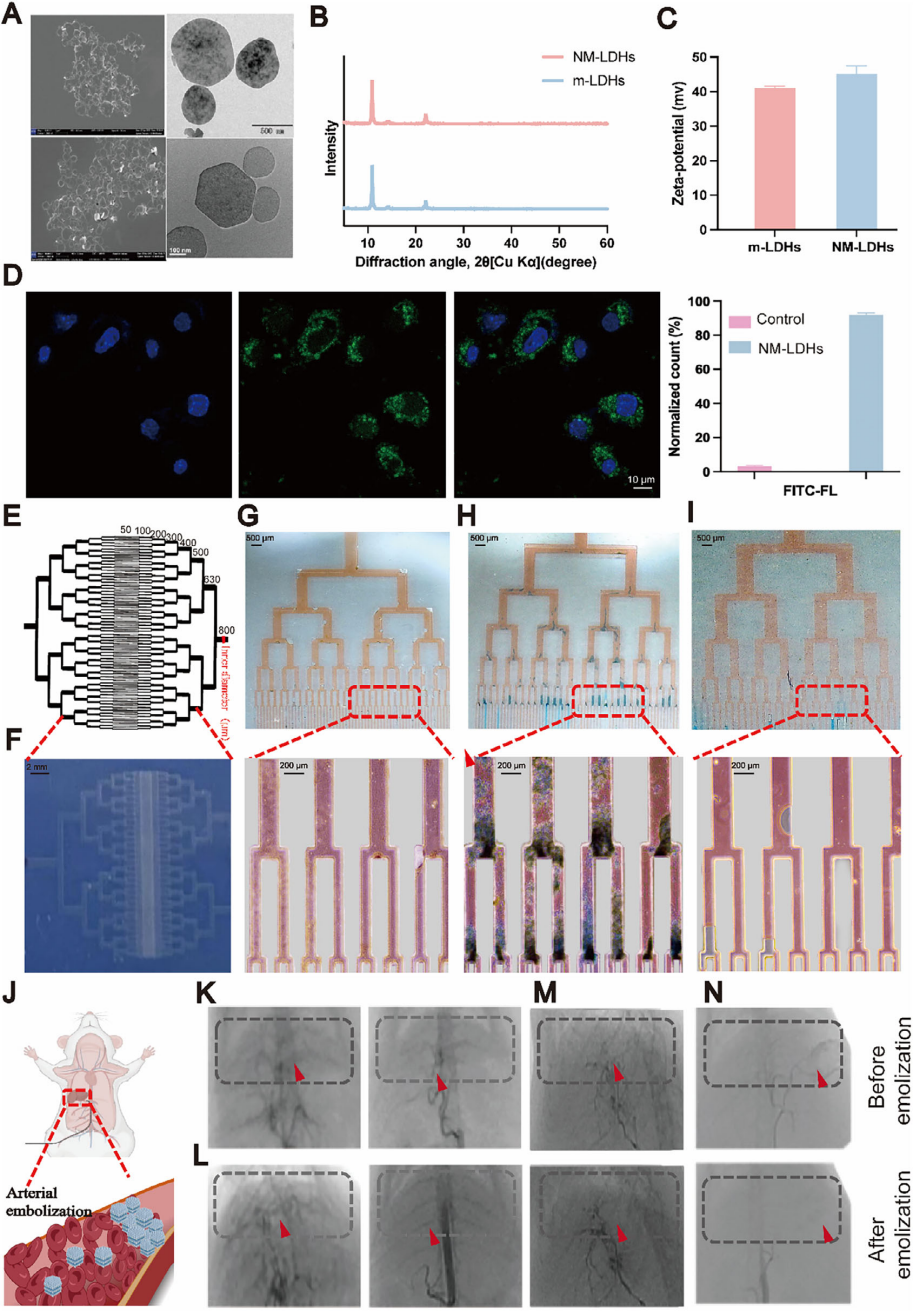
Characterization of LDHs with different particle sizes and their application in TAE. (A) The SEM images of m‐LDHs and NM‐LDHs. (B) XRD patterns of m‐LDHs and NM‐LDHs. (C) Zeta potential of m‐LDHs and NM‐LDHs. (D) Internalization of NM‐LDHs‐FITC by DC2.4 cells observed via confocal microscopy and flow cytometry. Scale bar: 10 µm. (E) Schematic of the microfluidic chip for in vitro vascular embolization modeling. (F) Verification of microfluidic chip patency by red ink injection Scale bar: 500 µm. (G–I) Microfluidic chip images before and after embolization with m‐LDHs (G), NM‐LDHs (H), and lipiodol (I). (J) Schematic diagram of X‐ray fluoroscopy evaluation for embolization efficacy of different agents. Scale bar: 500 µm (F–H) and 200 µm (I). (K–M) X‐ray fluoroscopy images of rat hepatic arteries before and after embolization with m‐LDHs (K), NM‐LDHs (L), and lipiodol (M). (N) X‐ray fluoroscopy images of orthotopic liver cancer model rats before and after NM‐LDHs embolization. Red arrows indicate the blood vessels before and after arterial embolization.

Layered double hydroxides (LDHs), owing to their strong surface electropositivity and tunable physicochemical properties, exhibit potential for enhanced ionic conductivity and aggregation‐driven precipitation in complex ionic environments such as blood. To explore their applicability in TAE, we synthesized two larger‐sized LDH nanosheets, m‐LDHs and NM‐LDHs. The structural and physicochemical properties of m‐LDHs and NM‐LDHs were thoroughly characterized, confirming their successful preparation. SEM analysis revealed that both materials exhibited regular hexagonal lamellar structures (Figure 2A), with average particle sizes of 0.45 ± 0.15 µm for m‐LDHs (Figure S2A) and 0.97 ± 0.27 µm (Figure S2B) for NM‐LDHs. TEM further validated their well‐defined crystalline structures, while XRD analysis displayed sharp characteristic diffraction peaks at d003 and d006 (Figure 2B), indicative of high crystallinity. FTIR spectra exhibited characteristic peaks of LDHs, including signals at 3500, 621, 776, 672, and 448 cm^−^^1^ (Figure S2C,D). DLS analysis demonstrated surface zeta potentials of +41.09 mV for m‐LDHs and +45.19 mV for NM‐LDHs (Figure 2C), underscoring their stability. These findings collectively confirm the successful synthesis of LDHs with distinct particle sizes and excellent physicochemical properties, which are suitable for further applications. We then studied the cell internalization of FITC‐labeled NM‐LDHs by co‐culturing with dendritic cells and macrophages. The internalization efficiency of NM‐LDHs was 91.9% in dendritic cells (Figure 2D), and it was also internalized by hepatocellular carcinoma cells (Figure S3A,B).

### 2.3 NM‐LDHs Succeed in Generating an Effective TAE

Alterations in the local electrostatic environment of charged particles induce smartly assemble and hierarchical stacking through charge‐mediated interactions. To simulate the dynamic embolization process of NM‐LDHs in vivo, an in vitro vascular embolization model was developed using microfluidic chip technology. This chip, featuring fractal microchannels with diameters ranging from 50 µm (the smallest pore size) to 800 µm (the largest), mimics the hierarchical vascular network of liver cancer (Figure 2E). Blue ink was initially injected to confirm patency (Figure S4A–C), followed by embolic agents, and red ink was introduced to evaluate occlusion under microscopy. Results indicated that m‐LDHs (Figure 2G) failed to occlude tumor vasculature effectively, due to their smaller particle size. In contrast, NM‐LDHs successfully occluded finer channels and demonstrated embolization performance a comparable to that of lipiodol (Figure 2G,I). Angiographic imaging under X‐ray fluoroscopy further evaluated the embolic capacity of different agents (Figure 2J). Selective catheterisation of the hepatic artery revealed no significant changes in blood flow following the use of s‐LDHs (Figure S4D,E) or m‐LDHs (Figure 2K). Conversely, NM‐LDHs effectively occluded the right and left hepatic branches, as evidenced by the absence of arterial flow post‐embolization (Figure 2L), showing a similar embolization efficacy to lipiodol (Figure 2M) (Figure 2M). Upon exposure to the bloodstream, NM‐LDHs undergo surface charge modulation, which facilitates their self‐assembly and hierarchical stacking. Concurrently, electrostatic interactions with negatively charged plasma proteins such as albumin and hemoglobin promote crosslinking and aggregation, ultimately yielding larger composite structures that function as efficient embolic agents. To evaluate the embolic efficacy of smartly assembled NM‐LDHs, we constructed an orthotopic liver cancer model in rats by injecting N1S1 cells. Hepatic angiography prior to embolization revealed prominent tumor neovascularization. Following NM‐LDHs injection, the tumor‐supplying arteries were no longer visible, confirming successful embolization (Figure 2N).

### 2.4 NM‐LDHs Mediate the Radiofrequency‐Responsive Heating Effect

**FIGURE 3:**
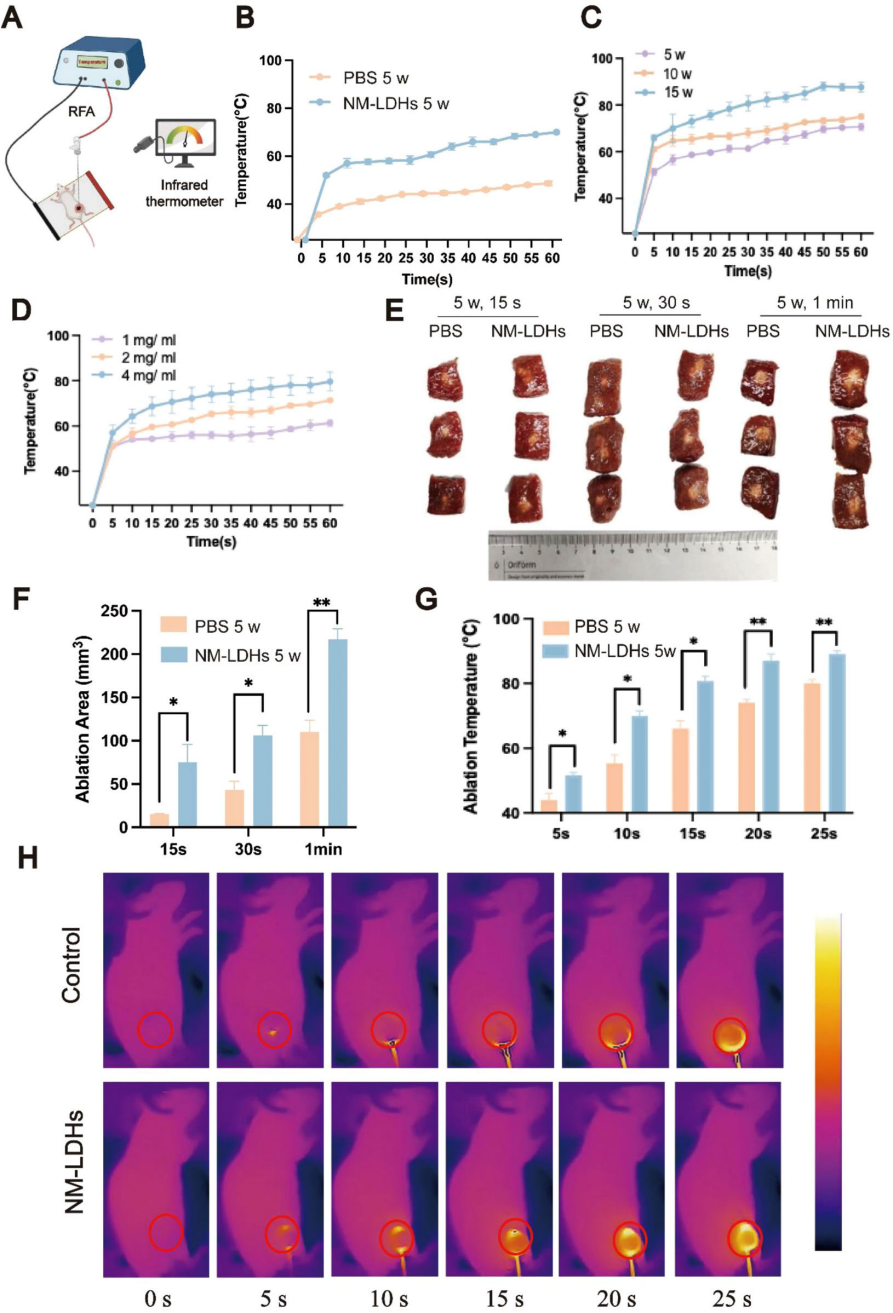
Thermal enhancement effect of NM‐LDHs under radiofrequency ablation. (A) Schematic diagram of the experimental setup for evaluating the thermal enhancement effect of NM‐LDHs under radiofrequency ablation. (B) Temperature‐time curves of PBS and NM‐LDHs (2 mg/mL) solutions under 5 W radiofrequency power. (C) Temperature‐time curves of PBS and NM‐LDHs (2 mg/mL) solutions under different radiofrequency power levels (5 W, 10 W, and 15 W). (D) Temperature‐time curves of NM‐LDHs solutions at different concentrations (1, 2, and 4 mg/mL) under 5 W radiofrequency power. (E,F) Tissue sections and statistical analysis of ablation volumes in fresh porcine liver tissue models after RFA at different time points (15, 30 s, and 1 min) for PBS and NM‐LDHs groups. (G) Temperature‐time curves of tumors in a mouse subcutaneous hepatocellular carcinoma model under 5 W radiofrequency power for PBS and NM‐LDHs groups. (H) Infrared thermal imaging of PBS and NM‐LDHs groups under 5 W radiofrequency power in a mouse subcutaneous hepatocellular carcinoma model. Data presented as mean ± s.d. All replicates are biological. Two‐tailed Student's *t‐*test was performed for statistical significance. ^*^ *P* < 0.05, ^**^ *P* < 0.01, ^***^ *P *< 0.001, ^****^ *P* < 0.0001, ns, not statistical significance.

Due to their highly positive charge, NM‐LDHs potentially respond to radiofrequency fields and amplify thermal ablation. In vitro, NM‐LDHs significantly increased the heating rate compared to PBS at 5 W (Figure 3A). Within 10 s, PBS temperature rose from 25°C to 39°C, while NM‐LDHs reached 57°C; after 1 minute, NM‐LDHs achieved 70°C versus 49°C for PBS (Figure 3B; Figure S5A). Further analysis showed that increasing the radiofrequency power accelerated the heating rate in the NM‐LDHs solution, highlighting the enhanced ionic oscillation (Figure 3C). NM‐LDHs concentration also affected heating: 4 mg/mL produced an 18.3°C increase after 1 minute, significantly more than 1 mg/mL (Figure 3D).

Post‐ablation, liver tissues showed a white necrotic zone with clear margins (Figure 3E). NM‐LDHs markedly enlarged ablation volumes versus PBS at all time points: 75.2, 106.1, and 216.9 mm^3^ versus 15.5, 43.5, and 110.2 mm^3^ at 15, 30, and 60 s, respectively (Figure 3F). In vivo, a mouse subcutaneous liver cancer model confirmed faster tumor heating with NM‐LDHs at 5 W, reaching 77.0°C at 15 s and 90.0°C at 25 s, compared to 66.3°C and 80.0°C with PBS (Figure 3G,H), confirming NM‐LDHs’ enhanced thermal effect during RFA.

### 2.5 NM‐LDHs Elicits Robust Anti‐Tumor Immune Responses

**FIGURE 4:**
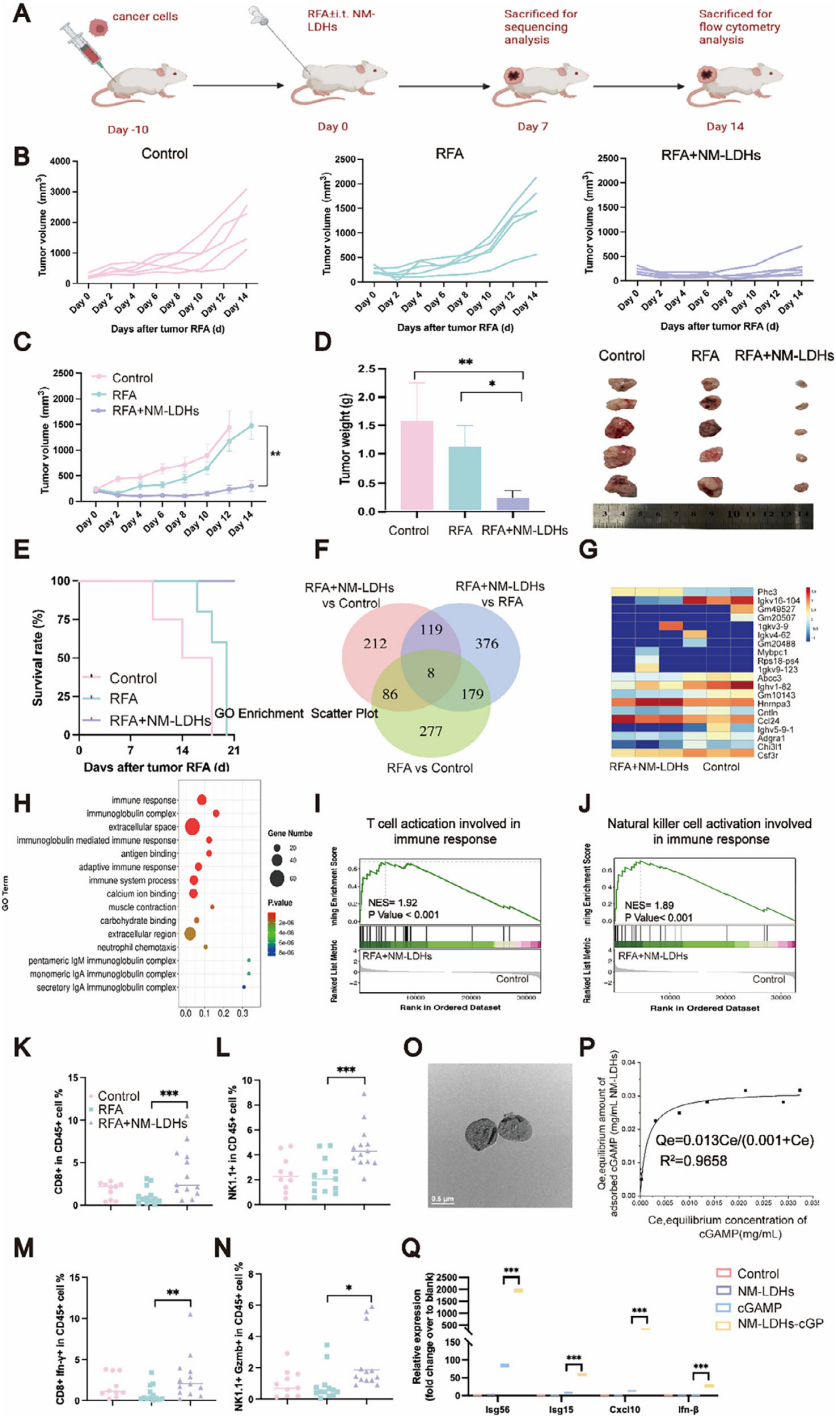
NM‐LDHs‐triggered anti‐tumor immune response and preparation of NM‐LDHs‐cGP. (A) Experimental flowchart for evaluating the therapeutic efficacy of RFA combined with NM‐LDHs in a mouse subcutaneous tumor model. (B–D) Average tumor growth curves (B), individual tumor growth curves (C), and photographs with weight statistics of excised subcutaneous tumors at 14 days post‐treatment (D) in mice (*n* = 5) receiving different treatments. (E) Survival period of tumor‐bearing mice after receiving different treatments (*n* = 5). (F) Venn diagram of gene expression from RNA‐seq results in tumor tissues of mice from three different treatment groups at 7 days post‐treatment (*n* = 3). (G) Heatmap showing the top 20 differentially expressed genes between the RFA+NM‐LDHs group and untreated mice. (H) GO enrichment analysis of the transcriptomics datasets. (I,J) GSEA analysis of pathway activity, including T cell and NK cell activation involved in immune response. The curves represent changes in the enrichment score (NES). P‐values and FDR values indicate statistical significance. (K,L) Quantification results of the percentage of CD8+ T cells (K) and NK cells (L) in tumors at 14 days post‐treatment (*n* = 13). (M,N): Quantitative analysis of the proportion of Ifnγ‐positive CD8+ T cells (M) and Granzyme B (GZMB)‐positive NK cells (N) in tumors at 14 days post‐treatment. (O) TEM images of NM‐LDHs‐cGP. Scale bar: 0.5 µm (P) Adsorption isotherm of cGAMP on NM‐LDHs fitted to the Langmuir equation. (Q) qPCR analysis of STING pathway activation in DC2.4 cells 4 h after different treatments. Data presented as mean ± s.d. All replicates are biological. Two‐way ANOVA, One‐way ANOVA or two‐tailed Student's *t*‐test was performed for statistical significance. ^*^ *P* < 0.05, ^**^ *P* < 0.01, ^***^ *P *< 0.001, ^****^ *P* < 0.0001, ns, not statistical significance.

The in vivo antitumor efficacy of combined RFA + NM‐LDHs was tested in a subcutaneous tumor‐bearing mouse model (Figure 4A). Tumor growth curves showed that RFA + NM‐LDHs significantly inhibited liver cancer progression (Figure 4B). Tumor volume in the RFA group rose from 242.21 to 1474.32 mm^3^, while in the combination group it was reduced to 299.04 mm^3^ by day 14 (Figure 4C). These results aligned with tumor weight data (Figure 4D). Moreover, all mice in the RFA + NM‐LDHs group survived to day 21, significantly longer than the RFA group (Figure 4E).

Transcriptomic sequencing (RNA‐seq) on tumors from nine mice identified 376 differentially expressed genes (Figure 4F). The top 20 up‐ and downregulated genes are shown in Figure 4G. GO and KEGG analyses revealed enrichment of immune‐related pathways, including “immune response,” “adaptive immune response,” and “NK cell‐mediated cytotoxicity” (Figure 4H; Figure S6A). GSEA showed upregulation of key immune pathways such as “T cell activation” (Figure 4I), “NK cell activation” (Figure 4J), and “positive regulation of NK cell‐mediated cytotoxicity” (Figure S6B) in the combination group versus control. These data strongly support enhanced NK and T cell–mediated immunity activation by RFA + NM‐LDHs.

On day 14 post‐treatment, compared to RFA alone, the proportion of CD8+ T cells within CD45+ immune cells in the RFA + NM‐LDHs group rose from 1.10% to 3.80% (Figure 4K; Figure S6C), and NK cells increased from 2.27% to 4.58% (Figure 4L; Figure S6D). Specifically, CD8+ T cells secreting IFN‐γ and NK cells secreting Granzyme B (GZMB) increased from 0.31% and 0.88% to 2.05% and 2.35%, respectively (Figure 4M; Figure S6E; Figure 4N; Figure S6F). The RFA‐only group showed mild, transient immune responses with no significant CD8+ or NK cell activation.

### 2.6 Synthesis of NM‐LDHs‐cGP and Its Immune Potentiation through cGAS‐STING Pathway Activation

To promote the anti‐tumor immune response post‐embolization or RFA, a multifunctional therapeutic platform NM‐LDHs‐cGP was further developed by incorporating NM‐LDHs and cGAMP, a STING agonist that could activate immune responses via cGAS‐STING pathway. NM‐LDHs‐cGP showed excellent dispersion and homogeneity, with a diameter of 0.99 ± 0.19 µm (Figure 4O). Moreover, the crystalline structure of NM‐LDHs remained stable after cGAMP loading, with only a slight reduction in zeta potential (Figure S7A,B). Langmuir adsorption analysis indicated that 1 mg of NM‐LDHs could adsorb up to 0.031 mg of cGAMP, reaching saturation at a 1:33 ratio (Figure 4P). Subsequently, a 1:50 cGAMP‐to‐NM‐LDHs ratio was adopted to preserve the NM‐LDHs' capacity to adsorb tumor‐associated antigens (TAAs). NM‐LDHs‐cGP at a concentration of 125 µg/mL achieves an exceptionally high cellular uptake efficiency exceeding 90% (Figure S7C). We next evaluated cGAS‐STING pathway activation in DC2.4 and Hepa1‐6 cells after NM‐LDHs‐cGP treatment. Compared to the free cGAMP group, NM‐LDHs‐cGP significantly enhanced the expression of IFN‐β, CXCL10, Isg15, and Isg56, highlighting the superior capacity of NM‐LDHs‐cGP to amplify downstream immune responses (Figure 4Q; Figure S7D). Furthermore, intratumoral validation confirmed that the loading of cGAMP exerts no significant impact on the intrinsic RF‐induced heating profiles of NM‐LDHs, ensuring robust thermal efficacy (Figure S7E).

### 2.7 Synergistic Effect of NM‐LDHs in Transarterial Immunoembolization (TAIE) and RFA

**FIGURE 5:**
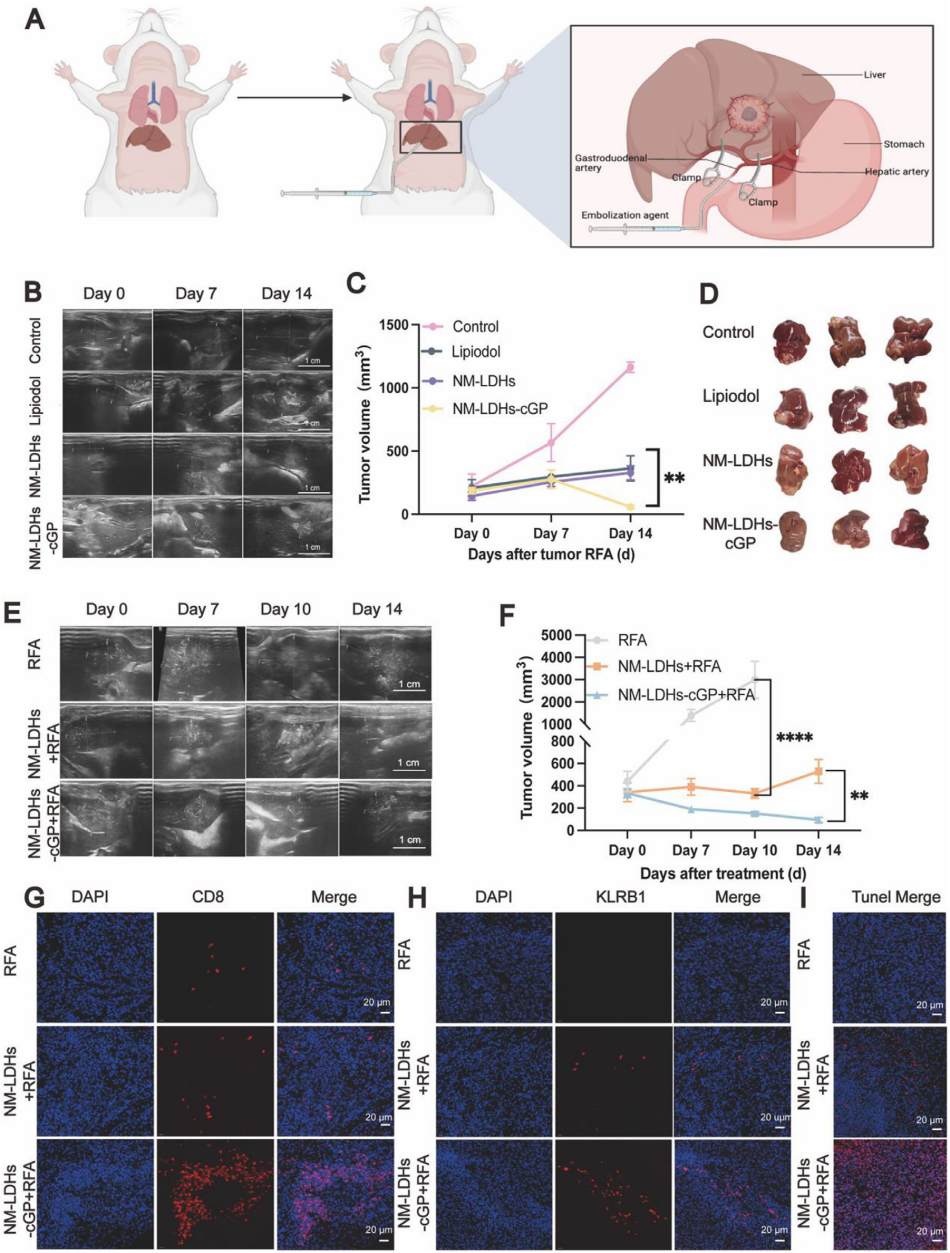
Synergistic effect of transarterial immunoembolization (TAIE) and RFA in the N1S1 rat orthotopic liver cancer model using NM‐LDHs‐cGP. (A) Schematic diagram of the experimental design. N1S1 tumor cells were implanted into the left liver lobe to establish an orthotopic rat hepatocellular carcinoma model, followed by retrograde hepatic artery catheterization via the gastroduodenal artery for embolization approximately two weeks later. (B–D) In the single arterial embolization experiment, representative ultrasound images at 7‐ and 14‐days post‐treatment (B), tumor volume change curves (C), and photographs of orthotopic liver tumors at 14 days post‐treatment (D) are shown (*n* = 4). Scale bar: 500 µm. (E,F) In the combined arterial embolization and RFA experiment, representative ultrasound images at 0‐, 7‐, and 14‐days post‐treatment (E) and tumor volume change curves (F) are presented (*n* = 4). Scale bar: 500 µm. (G–I) Immunofluorescence staining images of CD8^+^ T cell infiltration (G), NK cell infiltration (H), and TUNEL staining images of tumor cell apoptosis (I) in tumor tissues after treatment with RFA, NM‐LDHs+RFA, and NM‐LDHs‐cGP+RFA. Scale bar: 20 µm. Data presented as mean ± s.d. All replicates are biological. Two‐way ANOVA was performed for statistical significance.^*^ *P* < 0.05, ^**^ *P* < 0.01, ^***^ *P *< 0.001, ^****^ *P* < 0.0001, ns, not statistical significance.

Enhancing embolization therapy efficacy remains challenging. Smartly assembled NM‐LDHs‐cGP was evaluated as a transarterial immunoembolization (TAIE) agent in an in situ N1S1 tumor‐bearing rat model (Figure 5A, S8A). ICP‐OES confirmed the smartly assembled NM‐LDHs deposition of NM‐LDHs within the tumor (Figure S8B). On days 7 and 14 post‐TAIE, NM‐LDHs‐cGP significantly suppressed tumor growth by ultrasound compared to lipiodol‐only TAE (Figure 5B,C), consistent with tumor weight data (Figure 5D). TUNLE (Figure S9A,B) and Ki67 staining (Figure S9C,D) showed reduced proliferation and increased apoptosis in the NM‐LDHs‐cGP group.

Notably, substantial heat would be lost during RFA due to the rich vasculature in the side liver cancer (named “heat‐sink” effect). We further assessed the synergy of smartly assembled NM‐LDHs‐cGP‐based TAIE with RFA. RFA alone showed limited tumor inhibition (1163.84 mm^3^), moderate suppression occurred with NM‐LDHs+RFA (455.05 mm^3^), and NM‐LDHs‐cGP+RFA produced the strongest effect, reducing tumor volume to 143.27 mm^3^ at day 14 (Figure 5F). Histological and fluorescence analyses two weeks post‐treatment revealed markedly increased CD8+ T and NK cell infiltration in the NM‐LDHs‐cGP+RFA group (Figure 5G‐H; Figure S10A,B). TUNEL (Figure 5I; Figure S10C,D) and Ki67 staining (Figure S10E,F) confirmed extensive apoptosis and growth inhibition.

**FIGURE 6:**
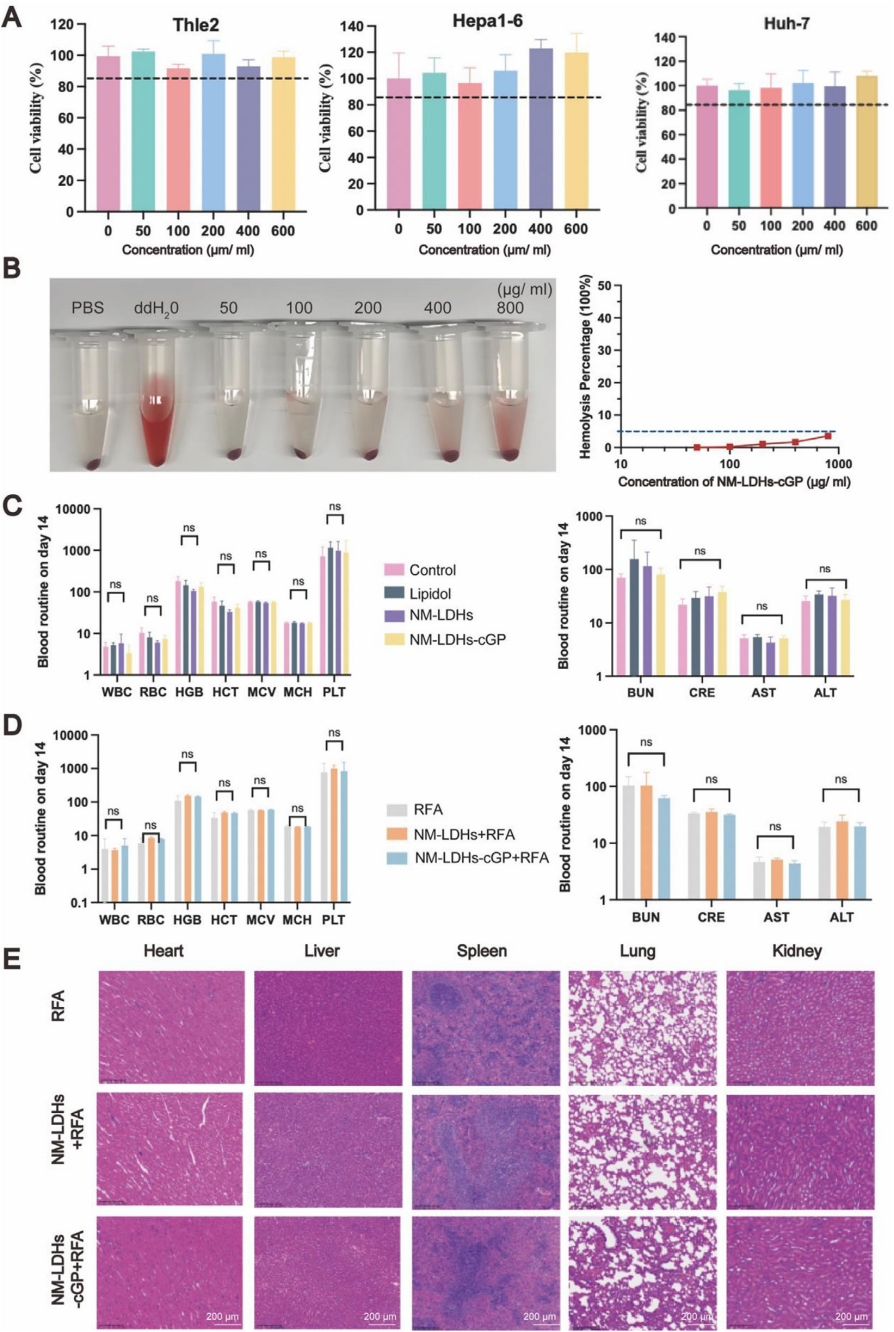
Biocompatibility assessment of NM‐LDHs and NM‐LDHs‐cGP. (A) Cell viability of Hepa1‐6 (mouse hepatocellular carcinoma cells), Huh‐7 (human hepatocellular carcinoma cells), and Thle‐2 (normal liver cells) after co‐incubation with different concentrations of NM‐LDHs‐cGP. (B) Hemolysis rate of murine red blood cells following incubation with different concentrations of NM‐LDHs‐cGP. (C) Complete blood count and blood biochemical analysis results in rats from the single arterial embolization experiment at 14 days post‐treatment. (D) Complete blood count and blood biochemical analysis results in rats from the combined arterial embolization and RFA experiment at 14 days post‐treatment. (E) Histopathological changes in major organs (heart, liver, spleen, lung, and kidney) across different treatment groups. Scale bar: 200 µm. Data presented as mean ± s.d. All replicates are biological. Two‐tailed Student's *t*‐test was performed for statistical significance. ^*^ *P* < 0.05, ^**^ *P* < 0.01, ^***^ *P *< 0.001, ^****^ *P* < 0.0001, ns, not statistical significance.

Biosafety evaluation showed no significant cytotoxicity of NM‐LDHs in Hepa1‐6, Huh‐7, or Thle‐2 cells (Figure 6A; Figure S11A–C). Furthermore, Hemolysis rates remained below 5% even at 800 µg/mL NM‐LDHs, indicating excellent hemocompatibility (Figure 6B). Additionally, Hematological and biochemical analyses showed no significant differences among groups (Figure 6C,D). Histopathology revealed no morphological or pathological abnormalities in major organs (Figure 6F; Figure S11D–G). These comprehensive results confirm the excellent in vitro and in vivo biosafety of NM‐LDHs and NM‐LDHs‐cGP.

### 2.8 NM‐LDHs‐cGP Enhances Immune Responses in Primary‐Cell Isolation From Patients

**FIGURE 7:**
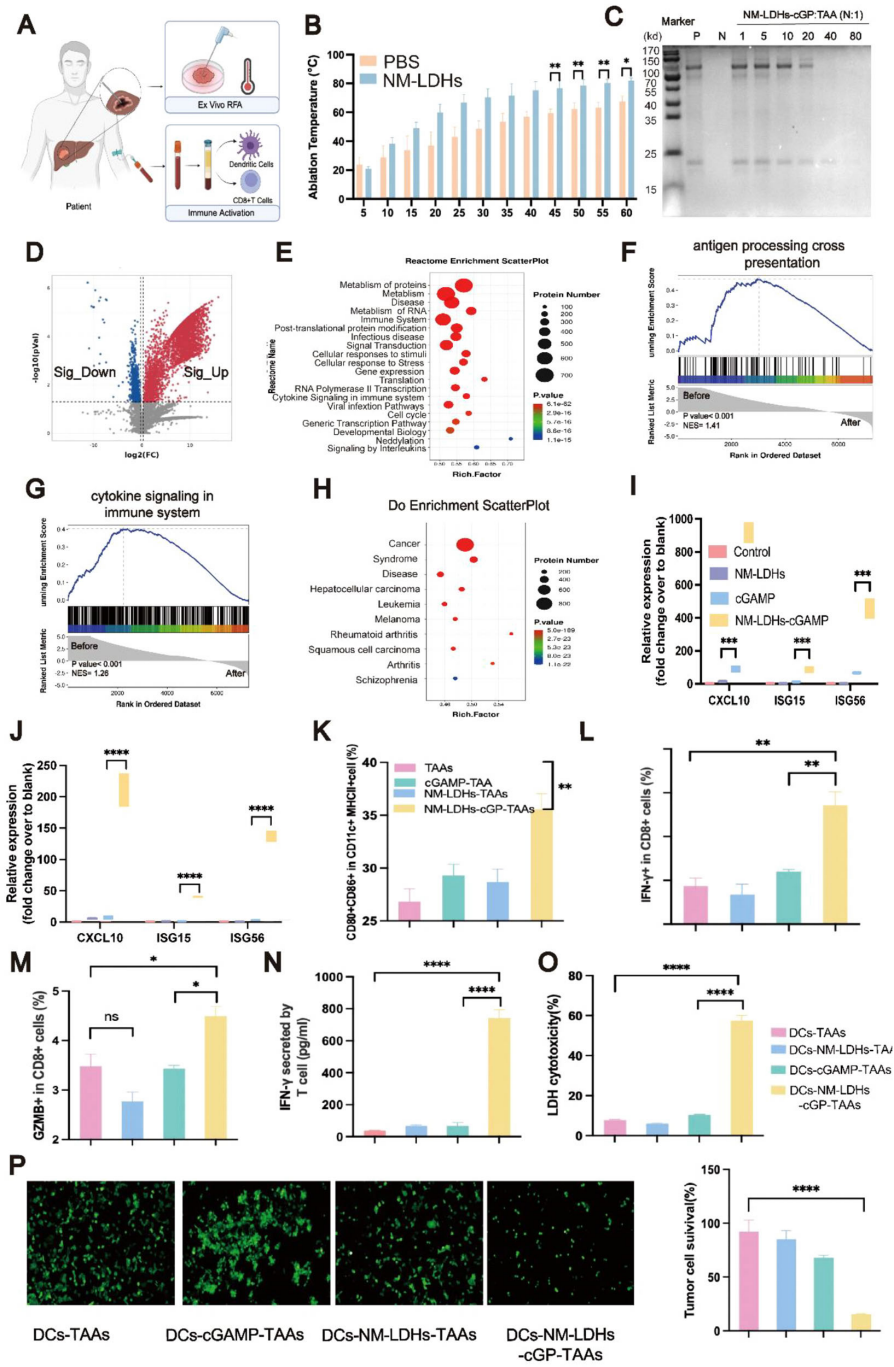
Synergistic Radiofrequency Thermal Enhancement and Immune Activation of NM‐LDHs‐cGP in Human Hepatocellular Carcinoma. (A) NM‐LDHs‐cGP mediated RF‐responsive heating and immune activation in human liver cancer system. (B) Temperature‐time curves of PBS and NM‐LDHs groups in human hepatocellular carcinoma tissues at different time points under RFA. (C) Qualitative analysis by SDS‐PAGE of NM‐LDHs‐cGP adsorption with human tumor antigens at different mass ratios. (D) Volcano plot of differentially expressed proteins in the supernatant before and after NM‐LDHs‐cGP adsorption (*n* = 3). The horizontal axis represents log2(FC), and the vertical axis represents ‐log10(P‐value). Red dots indicate upregulated proteins, and blue dots indicate downregulated proteins. (E) Reactome enrichment analysis of the proteomics datasets. (F,G) GSEA analysis of pathway activity for antigen processing and cross‐presentation (F) and cytokine signaling in the immune system (G) of proteins adsorbed by NM‐LDHs‐cGP. The curves represent changes in the enrichment score (NES). P‐values and FDR values indicate statistical significance. H, Disease Ontology (DO) analysis of the proteomics datasets. (I,J) qPCR analysis of STING pathway activation in peripheral blood mononuclear cells (PBMCs) and Huh‐7 cells after different treatments. (K) Flow cytometry analysis of CD80 and CD86 expression in human dendritic cells stimulated with LDHs‐cGAMP‐TAAs for 24 h (*n* = 3). (L,M) Flow cytometry analysis of IFN‐γ+ (L) and GZMB (M) expression in human T cells co‐cultured with differently treated human dendritic cells for 7 days (*n* = 3). (N) Secretion levels of IFN‐γ+ in the culture supernatant after co‐culturing human T cells with differently treated human dendritic cells for 10 days (*n* = 3). (O,P) Cytotoxic effect on Huh‐7 cells induced by differently treated T cells assessed by LDH assay (O) and fluorescence imaging (P). Scale bar: 200 µm. Data presented as mean ± s.d. All replicates are biological. Two‐tailed Student's *t*‐test or One‐way ANOVA was performed for statistical significance.^*^ *P* < 0.05, ^**^ *P* < 0.01, ^***^ *P *< 0.001, ^****^ *P* < 0.0001, ns, not statistical significance.

To assess the clinical potential of NM‐LDHs‐cGP, human liver cancer was used to evaluate its RF heating and immune activation capabilities. In RF heating experiments, NM‐LDHs‐cGP rapidly reached 81.83°C within 1 min, significantly exceeding the PBS group (67.50°C), confirming its superior clinical heating performance (Figure 7A).

We next investigated the adsorption capacity of NM‐LDHs‐cGP for tumor‐associated antigens (TAAs). Using BSA as a model antigen, complete adsorption was achieved at a 5:1 NM‐LDHs:BSA mass ratio (Figure S12A,B). When loaded with cGAMP, NM‐LDHs captured TAAs from HCC tissues at an optimized 50:1:2.5 ratio (NM‐LDHs:cGAMP:TAAs) (Figure 7B,C). Proteomic analysis identified 3,623 bound proteins (Figure 7D), including DAMPs like HSPs and HMGB1 (Figure S13A,B), which are key for antigen cross‐presentation and immune activation. Reactome analysis revealed enrichment of immune‐related pathways, including cytokine signaling (R‐HSA‐1280215), interleukin signaling (R‐HSA‐449147), and viral infection (R‐HSA‐9824446), all associated with anti‐tumor immunity (Figure 7E). Furthermore, GSEA confirmed activation of antigen processing and cytokine signaling (Figure 7F,G), and DO analysis linked these proteins to HCC (Figure 7H), supporting NM‐LDHs’ ability to enhance anti‐HCC responses. To test immune activation in human cells, experiments were performed using Huh‐7 cells and PBMCs. Compared to free cGAMP, NM‐LDHs‐cGP significantly upregulated ISG56, ISG15 and CXCL10 (Figure 7I,J). Additionally, flow cytometry analysis of peripheral blood dendritic cells (PBDCs) showed that NM‐LDHs‐cGP, loaded with patient‐derived TAAs, significantly promoted DC maturation and upregulated co‐stimulatory molecules CD80 and CD86 (Figure 7K; Figure S13C).

Further, CD8^+^ T cell proliferation and activation were assessed after 7‐day co‐culture with treated PBDCs. The NM‐LDHs‐cGP‐TAAs group showed the highest proliferation (Figure S14A), and flow cytometry revealed a shift toward an effector phenotype with increased IFN‐γ+ (Figure 7L; Figure S14B,C) and GZMB+ CD8+ T cells (Figure 7M; Figure S14D). Cytokine assays confirmed elevated IFN‐γ (Figure 7N) and TNF‐α (Figure S15A). In co‐cultures with GFP+ Huh‐7 cells, NM‐LDHs‐cGP‐TAAs induced potent cytotoxicity, with a 5.52‐fold increase in LDHA release (Figure 7O) and 77.01% reduction in GFP signal (Figure 7P).

To summarize, NM‐LDHs‐cGP exhibited potent immune activation and anti‐tumor effects, enhancing immune cell maturation and promoting CD8+ T cell activation. These results underscore its potential as an effective therapeutic strategy for liver cancer, combining immune modulation with targeted tumor cell elimination.

## 3 Discussion

Despite advances in oncology, the 5‐year survival rate for hepatocellular carcinoma (HCC) has remained largely unchanged over the past few decades [25]. While the combined application of radiofrequency ablation (RFA) and transarterial embolization (TAE) may improve clinical outcomes, the low immunogenicity of HCC and the immunosuppressive tumor microenvironment (TME) continue to hinder the efficacy of conventional therapies [26]. In this study, we developed a novel TAIE agent, smartly assembled near‐micron‐scaled layered double hydroxide nanoparticles (NM‐LDHs‐cGP), for enhancing the efficiency of RFA and embolization, modulating the tumor microenvironment, and boosting systemic antitumor immunity against HCC.

Microspheres, widely used as solid embolic agents in clinical practice, have increasingly been engineered into multifunctional composites by incorporating nanoparticles such as ​Fe_3_O_4_ [27], hydrogels [28], and hydroxyapatite [29]. However, their relatively large particle size limits their ability to fully occlude the disorganized microvascular network at the tumor periphery [30, 31]. Layered double hydroxides (LDHs) with tunable properties were synthesized in larger sizes (m‐LDHs: 0.45 ± 0.15 µm; NM‐LDHs: 0.97 ±0.27 µm). Owing to their dynamic responsiveness to local charge fluctuations and microenvironmental variations, NM‐LDHs can smartly assembled into stable vascular embolic agents, enhancing TAE efficacy. Despite their near‐micron size, NM‐LDHs exhibited efficient cellular internalization in immune (about 90%) and cancer cells, supporting their potential as an effective delivery and adjuvant platform. As for the embolization performance, smartly assembled NM‐LDHs effectively occluded tumor vessels and demonstrated successful embolization in a microfluidic chip model and an orthotopic rat liver cancer model (Figure 2H,L,N).

RFA encounters significant limitations when treating irregular or large tumor nodules [32, 33]. The novel NM‐LDHs platform addresses these challenges by demonstrating superior radiofrequency thermal responsiveness. Multiple models (in vitro, murine and ex vivo) confirm NM‐LDHs' superior heating efficiency at lower doses and RF power. Crucially, the system exhibits dose‐dependent thermal performance, with both heating kinetics and ablation volumes scaling proportionally with increased NM‐LDHs concentration and applied RF power.The mechanisms by which RFA contributes to early HCC recurrence and immune responses remain unclear. Most human data on RFA‐induced immunity come from exploratory analyses of peripheral blood [34, 35]. Comprehensive tumor tissue analysis is essential for improving RFA treatment in liver cancer. We designed a prospective clinical trial to obtain adjacent incompletely ablated HCC tissues during RFA using biopsy needles. Proteomic profiling characterizes molecular and signaling pathway changes in the tumor microenvironment (TME) induced by incomplete RFA. Our findings show that residual tumor tissue after RFA shifts toward an immunosuppressive microenvironment, marked by reduced antigen processing and diminished activation of T and NK cells.

Adjuvants are vital in vaccines for enhancing immune responses [36, 37]. Since 1926, aluminum‐based adjuvants (alum) have been the most widely used [38]. However, conventional alum often fails to induce strong immunostimulant due to limited local response, incompatibility with some antigens, and inability to trigger CD8+ T cell‐mediated immunity [37, 38]. Beyond enhancing TAE and RF‐induced heating, NM‐LDHs—composed of Mg and Al elements—serve as an ideal immune adjuvant for combination therapy, capable of eliciting systemic specific immunity through both humoral and cellular immune responses. Transcriptomic and flow cytometry analyses show that smartly assembled NM‐LDHs exhibit strong adjuvant‐like properties, potentially activating pathways such as “T cell activation,” “NK cell activation,” and “immune system process.” Following RFA, NM‐LDHs initiate a rapid innate immune response by recruiting NK cells to the tumor site, promoting nonspecific tumor cell attack and increased exposure of tumor antigens. Subsequently, in the presence of exposed tumor antigens, NM‐LDHs can trigger a robust tumor‐specific adaptive immune response to target residual local tumors. Concurrently, their capacity to modulate the immunosuppressive TME further enhances antitumor immunity.

The cGAMP compound acts as a STING agonist, activating the cGAS‐STING pathway to stimulate type I interferon production [39]. Studies have shown that LDHs, once internalized by antigen‐presenting cells (APCs), efficiently escape endosomes, enabling controlled cytoplasmic release of therapeutic agents [21, 40]. Our data show that the NM‐LDHs‐cGP nanocomposite delivers cGAMP more efficiently to neoplastic cells and APCs than free cGAMP, resulting in markedly enhanced IFN‐I pathway activation. In clinical oncology, TAE and TACE are established locoregional therapies with proven efficacy [41]. However, clinical data show that even repeated TAE/TACE often fail to fully eradicate tumors [42]. In this study, we applied smartly assembled NM‐LDHs‐cGP in TAIE procedures following standard TAE/TACE protocols. Comparative analyses reveal that smartly assembled NM‐LDHs‐cGP‐mediated TAIE achieves superior antitumor efficacy compared to conventional lipiodol‐based embolization. Previous clinical studies by Yao et al. showed that TACE combined with RFA yields better long‐term survival than RFA alone [43]. This synergy likely results from dual mechanisms: tumor volume reduction and mitigation of the heat‐sink effect via pre‐ablation embolization, thereby enhancing thermal‐induced tumor necrosis. Research by Li et al. developed cisplatin‐conjugated poly(N‐isopropylacrylamide‐co‐acrylic acid) (Pt‐PNAs) nanogels as radiofrequency‐responsive embolic agents [44]. Conventional chemotherapy is limited by non‐specific cytotoxicity, adverse effects, and transient responses [45, 46]. In contrast, immunomodulatory strategies targeting immunosuppression or enhancing immune activation show promise for inducing durable antitumor effects [47, 48]. Our study shows that combining smartly assembled NM‐LDHs‐cGP‐based TAIE with RFA significantly promotes intratumoral infiltration of CD8+ T cells and NK cells, while enhancing their tumoricidal activity.

Motivated by promising preclinical results, we extended our study to human‐derived liver cancer tissues and PBMCs to evaluate the translational potential of NM‐LDHs‐cGP. Consistent with animal studies, the platform improved RF heating in human tumors and captured tumor‐associated antigens, including key DAMPs like HSPs and HMGB1. Notably, NM‐LDHs‐cGP activated the STING pathway in patient‐derived immune cells, enhanced dendritic cell maturation, and promoted CD8^+^ T cell proliferation and cytotoxic differentiation. These findings underscore the strong clinical promise of the radiofrequency‐responsive approach to transarterial embolization–immunothermal therapy in liver cancer.

## 4 Experimental Section/Methods

### 4.1 Human Hepatocellular Carcinoma Sample

Fresh liver cancer tissue specimens were obtained from the Fourth Affiliated Hospital of Zhejiang University with an approval number of K2024148. In accordance with local ethical guidelines (as stipulated by the Declaration of Helsinki) and protocols approved by the institutional review board of the Fourth Affiliated Hospital of Zhejiang University, all fresh liver cancer tissue samples were anonymized and assigned unique codes. The experimental design was briefly described as follows. Six patients meeting the diagnostic criteria for HCC were enrolled in this prospective study. The study employed an iRFA protocol, with a 1 cm ablation margin for 5 min. The RFA system consisted of a 105‐watt generator and a length‐adjustable 16‐gauge electrode (LDRF‐120S, Lead Electron Corporation, Mianyang, China). Adjacent HCC tissue, incomplete ablated, was sampled using a 16‐gauge biopsy needle while maintaining the electrode tip temperature below 37°C for 30 min. Following this sampling, a complete ablation was performed, lasting 15 min. To ensure precision in RFA execution and tumor tissue sampling, the tumor specimens were collected with diameters between 3 and 5 cm and located within 1 cm of the liver surface. All RFA procedures were conducted under direct laparoscopic visualization. Fresh tissue samples were promptly preserved in liquid nitrogen for subsequent transcriptomic and proteomic analyses.

### 4.2 Cell Line and Animals

Mouse liver cancer Hepa1‐6 (CVCL_0327), human HCC Huh7 (CVCL_0336), and human immortalized hepatic epithelial THLE‐2 (CVCL_3803) cells were kindly provided as gifts by the Department of Hepatobiliary and Pancreatic Surgery, Hangzhou First People's Hospital, China, originally sourced from the Cell Bank of the Chinese Academy of Sciences (Hepa1‐6 and Huh7) and Procell Life Science & Technology (Thle‐2). RAW264.7 (CVCL_0336) was obtained from iCell Bioscience Technology Co., Ltd. (Shanghai, China), DC2.4 (CVCL_J409) was obtained from Fuheng Biology Technology Co., Ltd. (Shanghai, China) and rat HCC cells N1‐S1 (CVCL_3551) were obtained from the American Type Culture Collection (Manassas, VA, USA). Cells were tested negative for mycoplasma infection using the Mycoplasma Detection Kit (Beyotime, #C0297S). Sprague‐Dawley rats and C57BL/6 mice aged 6–8 weeks were purchased from Hangzhou Qizhen Laboratory Animal Technology Co. Ltd. The animal feeding and experimental protocols involved in this study were reviewed and approved by the Laboratory Animal Ethics Committee of Zhejiang University prior to the commencement of the research.

### 4.3 RNA‐seq Analysis and Data Processing

The total RNA of the residual tumor tissues was extracted by TRIzol. RNA purity and integrity were tested using NanoDrop ND‐1000 (NanoDrop, Wilmington, DE, USA) and Bioanalyzer 2100 (Agilent, CA, USA). After mRNA enrichment and fragmentation, the strand‐specific library was constructed, and the RNA libraries were sequenced on the Illumina NovaseqTM 6000 platform by LC Bio Technology CO., Ltd (Hangzhou, China). Then filter the data after the test to obtain clean data, compare the data to the murine source's reference genome, and conduct differentially expressed gene analysis, etc.

### 4.4 Proteogenomic Analysis

Statistical analysis was performed in R (version 4.0.0). The raw protein intensity will be normalized by method “medium”, Hierarchical clustering was performed using pheatmap package. Principal component analysis (PCA) was performed using metaX package. Correlation analysis was performed by Pearson correlation coefficient of cor package. T test was used for statistical differential analysis and a cut of p‐value ≤ 0.05 and fold change >1.2 was used to select statistically differential expressed proteins. Hypergeometric‐based enrichment analysis with KEGG Pathway, Gene Ontology and Reactome Pathway were performed to annotate protein sequences, individually. The software GSEA (v4.1.0) and MSigDB were used for gene set enrichment analysis to determine whether a set of genes in a specific GO term, KEGG pathway, DO term(human), Reactome (some model animals) in different situations. Meeting this condition |NES|>1, NOM p‐val<0.05, FDRq‐val<0.25 were considered to be significantly different between the two groups.

### 4.5 Preparation and Characterizations of NM‐LDHs

Mg‐Al‐Cl layered double hydroxides (LDHs) of varying particle sizes were synthesized using a co‐precipitation and hydrothermal method. Briefly, an alkaline solution containing 0.45 M NaOH was mixed with a salt solution comprising 0.7 M MgCl_2_ and 0.3 M AlCl_3_·6H_2_O under vigorous stirring. The resulting precipitate was collected, thoroughly washed, and initially heated at 100°C. Subsequently, the pretreated mixture underwent hydrothermal treatment at 150°C or 200°C for 2–3 days to enable controlled thermal growth, yielding LDHs with tunable particle sizes. To prepare LDHs‐FITC, the synthesized LDHs were mixed with FITC at a mass ratio of 50:1 and stirred for 1 h. The mixture was then washed once with deionized water and subjected to hydrothermal treatment. The resulting LDHs‐FITC product was stored at 4°C in the dark to protect it from light.

The surface charge (Zeta potential) of LDHs and NM‐LDHs‐cGP was analyzed using DLS. Particle size and morphology were examined through TEM and SEM. FTIR was performed using a Bruker vector FTIR spectrometer to analyze the infrared spectra of the samples. Additionally, XRD analysis was conducted over a scanning range of 5–90° to identify characteristic diffraction peaks and determine the lattice structure of the samples.

### 4.6 Cellular Uptake of NM‐LDHs

Confocal laser scanning microscopy (CLSM) was utilized to qualitatively evaluate the cellular uptake of NM‐LDHs. Hepa1‐6, DC2.4 and RAW264.7 cells were seeded into confocal culture dishes at a density of 2×10^5^ cells per well and incubated with FITC‐labeled NM‐LDHs for 4 h after a 24‐h seeding period. Cell nuclei were labeled with Hoechst 33342. The intracellular localization of the LDHs was visualized using an LSM780 confocal laser scanning microscope. DAPI was excited at 405 nm and emission was collected at 420–480 nm. FITC was excited at 488 nm and emission was collected at 500–550 nm.

Flow cytometry was used to quantitatively analyze the cellular uptake of L‐LDH. Hepa1‐6, DC2.4, and RAW264.7 cells were seeded in 12‐well plates at a density of 5 × 10^5^ cells per well and cultured at 37°C with 5% CO_2_ for 24 h. FITC‐labeled LDHs were incubated with the cells for 4 h. The medium was then removed, and the cells were washed 2–3 times with PBS. After trypsinization, the cells were collected and subjected to flow cytometry analysis.

### 4.7 Construction of an In Vitro Embolization Simulation Device

To evaluate the embolization effect of LDHs with varying particle sizes in vitro, a microfluidic chip‐based device simulating a tumor vascular network was designed. The microvascular simulation chip was prepared as follows: Polydimethylsiloxane (PDMS) and curing agent were mixed at a weight ratio of 10:1, stirred thoroughly for 30 min, and then poured onto a mold. The mold contained a 9‐level hierarchical channel design, with channel widths of 800, 630, 500, 400, 300, 200, 100, and 50 µm, and a channel height of 50 µm. The mixture was cured at 60°C for 2 h. The solidified substrate was then cut out with a blade, and holes were punched at designated positions using a hole puncher. The cured substrate was bonded to glass and placed in an oven at 50°C for 30 min to ensure complete adhesion, resulting in a microvascular simulation chip.

A syringe was used to inject 0.1 mL of LDHs and Lipiodol into the microvascular simulation area. The embolization effect at different hierarchical levels was visually observed and examined under a microscope to assess the peripheral embolization performance of the agents.

### 4.8 Interventional Hepatic Artery Embolization Experiment

Rats underwent hepatic and renal artery embolization via femoral artery catheterization. After fasting and water deprivation for 12 h, rats were anesthetized with an intraperitoneal injection of sodium pentobarbital (30 mg/kg). Once pain reflexes disappeared (5–10 min), the inguinal region was shaved, disinfected, and a 2–3 cm incision was made. The femoral artery was carefully isolated using forceps, and the distal end was ligated. A 2 mL syringe needle was used to puncture the femoral artery, and a microguidewire was inserted through the puncture site, followed by the placement of a 1.5F catheter over the guidewire, which was gradually advanced proximally. Under direct observation using digital subtraction angiography (DSA), the catheter was positioned into the hepatic or renal artery, and iodixanol was injected to visualize the arteries. Lipiodol and LDHs of different particle sizes were then injected at a rate of 1 mg/min. Five minutes after the injection of LDHs, iodixanol was injected again to visualize the arteries and evaluate the embolization effect. Three weeks post‐procedure, the rats' kidneys were harvested to assess the long‐term embolization effect.

### 4.9 Radiofrequency‐Responsive Heating Experiments In Vitro and In Vivo

#### 4.9.1 In Vitro Experiment

NM‐LDHs were diluted to varying concentrations using phosphate‐buffered saline (PBS), with a total sample volume of 5 mL for each group. The RF‐responsive heating effect of NM‐LDHs was investigated under different RF ablation powers and L‐LDH concentrations. An ablation electrode was connected, and the RF ablation probe was fully submerged below the liquid surface. The instrument was activated, and the ablation temperature was recorded at regular time intervals.

In ex vivo pig liver tissues, 2 mg of the sample solution was injected into the liver. The RF ablation power was set to 5 W. After the ablation was completed, the liver was cut along the insertion path of the RF probe to expose the cross‐section. The ablation effect was observed, and the ablation area was approximated as an ellipse. The long and short diameters of the elliptical ablation zone were measured, and the ablation area was calculated. The ablation volume (V) was estimated using the formula:


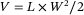

where 𝐿 is the long diameter, and 𝑊 is the short diameter of the ablation zone.

#### 4.9.2 In Vivo Experiment

For subcutaneous tumor‐bearing mice, RF ablation was initiated when tumor volumes reached 800–1000 mm^3^. Under 1.5% isoflurane anesthesia, the mice were placed on an electrode plate. 600 µg of the NM‐LDHs (10 µg/µL) was injected into the tumor. The RF ablation probe was inserted into the tumor center along its long axis, and temperature changes at the injection site were monitored in real time.

### 4.10 Animal Models

All animal experiments were conducted according to animal ethics policies that were approved by the Laboratory Animal Welfare Ethics Review Committee of Zhejiang University (ZJU20240764). In the subcutaneous (S.C.) tumor model, the fur on the right dorsal side of C57BL/6 mice was shaved, and 2 × 10^6^ Hepa1‐6 liver cancer cells were implanted subcutaneously. Approximately 10 days later, when the tumor diameter reached 6–8 mm, the mice were randomly divided into three groups: (I) blank, (II) RFA, (II)RFA+NM‐LDHs (600ug NM‐LDHs). For radiofrequency ablation (RFA) treatment, under 1.5% isoflurane anesthesia, the mice were placed on an electrode plate in a prone position. The RFA instrument (MedSphere, Shanghai, China) was connected, and a 17‐gauge monopolar electrode was inserted along the long axis of the tumor into the tumor center. The parameters were set to 5 W, with ablation starting after 10 s. For the RFA+NM‐LDHs treatment group, NM‐LDHs were immediately injected into the tumor after RFA treatment. Tumor volume and body weight were assessed every two days until the end of the observation period. The tumor volume was calculated using the formula:


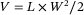

where 𝐿 is the long diameter, and 𝑊 is the short diameter of the tumor.

### 4.11 The Preparation of NM‐LDHs

To prepare 2'3'‐cGAMP‐loaded NM‐LDHs (NM‐LDHs‐cGP), 2'3'‐cGAMP was mixed with NM‐LDHs at varying mass ratios (ranging from 1:1 to 1:200). The NM‐LDHs were gradually added dropwise into the cGAMP solution under vertexing for 2 mins. The supernatant from the mixture was collected, and the free cGAMP content was quantified using a Nanodrop spectrophotometer (Thermo Fisher, USA). Finally, the adsorption isotherm of cGAMP on NM‐LDHs was modeled using the Langmuir equation:

$$\mathrm{Qe}=\mathrm{bQmCe}/\left(1+\mathrm{bCe}\right)$$

Qe represents the amount of cGAMP adsorbed per unit mass of LDHs at equilibrium (mg/mg LDHs). b is the adsorption equilibrium constant (mL/mg). Qm is the maximum adsorption capacity (mg/g). Ce is the equilibrium concentration of cGAMP in the solution (mg/mL).

### 4.12 In Vitro BMDC Activation

Bone marrow‐derived dendritic cells (BMDCs) were extracted from BALB/C mice. The femurs and tibias of the mice were isolated, and the bone marrow cavity was repeatedly flushed with medium to collect cells, using a filter to remove bone fragments and muscle. The cell suspension was then resuspended and added to complete RPMI‐1640 medium containing 10% FBS, with 20 ng/mL GM‐CSF and 10 ng/mL interleukin‐4. The medium was changed every two days. The non‐adherent are immature BMDCs on the 7th day.

BMDCs were seeded into 12‐well plates at a density of 1 million cells per well. After 24 h, the complete medium was replaced with serum‐free RPMI 1640. The BMDCs were then incubated with NM‐LDHs (125 µg/µL), cGAMP (2.5 µg/µL), or NM‐LDHs/cGAMP (NM‐LDHs: 125 µg/µL, cGAMP: 2.5 µg/µL) for 4 h. After incubation, the cells were collected and stained with various antibodies. Flow cytometry analysis was performed using BD LSR Fortessa flow cytometer (BD Biosciences, NJ, USA), and data were analyzed using FlowJo software (Tree Star, Ashland, OR).

### 4.13 Flow Cytometry

The method for preparing a single‐cell suspension has been previously described. Briefly, 14 days after different treatments, the mice were euthanized, and the tumors were excised and cut into small pieces for digestion and resuspension. The resulting tumor tissue suspension was then filtered through a 70 µm nylon mesh filter. Zombie Aqua Fixable Viability Kit was used for membrane staining, followed by incubation with various flow cytometry antibodies. For intracellular staining, cells were first stimulated with the Cell Activation Cocktail, followed by surface staining. The cells were then fixed, permeabilized, and incubated with antibodies against intracellular molecular markers. Finally, the samples were filtered again using a 40 µm nylon filter and analyzed by BD LSR Fortessa flow cytometer (BD Biosciences, NJ, USA). Data were analyzed using FlowJo software (Tree Star, Ashland, OR).

All antibodies and reagents used in flow cytometry were from BD Pharmingen or BioLegend. Including Zombie Aqua Fixable Viability Kit, Cell Activation Cocktail (with Brefeldin A), Fixation buffer, Permeabilization wash buffer, APC‐Cy7‐antimouse‐CD45, APC‐ntimouse‐CD3, BV42‐antimouse‐CD4, AF700–antimouse‐CD8, Percp‐Cy5.5‐antimouse‐NK1.1, BV421‐antimouse‐CD80, Percp‐Cy5.5‐antimouse‐CD86, PE‐Cy7‐antimouse‐IFN‐γ, PE‐antimouse‐Gzmb.

### 4.14 RNA Extraction and RT‐qPCR

Total RNA was extracted using TRIzol Invitrogen, Carlsbad, USA). The RNA was then reverse transcribed using a reverse transcription kit (TakaRa, Japan) for PCR reactions. Quantitative PCR was performed using SYBR Green Master Mix (Invitrogen, Carlsbad, USA). The relative expression fold was calculated using the 2^−ΔΔCt^ equation. Primer sequences of target genes are provided in Supplementary Table 1.

### 4.15 Rat Hepatoma Model

Sprague‐Dawley rats were used in this study. Animals were housed in cages with free access to food and water. The laboratory maintained a 12‐h light/dark cycle, and the room temperature was 25°C. All animal experiments were approved by the Institutional Animal Care and Use Committee and conducted following the basic guidelines for animal experiments and related activities at Zhejiang University. Surgical procedures were performed under isoflurane anesthesia. An orthotopic liver cancer model was established in rats under minilaparotomy. After anesthesia, the rats were placed in a supine position, and a small incision was made in the abdominal skin to expose the left lobe of the liver. Then, 2.5–3 × 10^6^ N1S1 cells dissolved in 50 µL phosphate‐buffered saline and 50 µL Matrigel were injected subcapsularly into the liver. After injection, the site was manually compressed for 4–5 min to prevent backflow of the injected cells. Finally, the skin was sutured, and the rats were allowed to recover. The growth of implanted tumors was monitored using ultrosonography.

### 4.16 Hepatic Artery Embolization and Radiofrequency Ablation

Once the implanted liver tumor reached a volume of 250 mm^3^, the rats were divided into the following groups: control, iodized oil embolization (2 mg), NM‐LDHs embolization (2 mg), and NM‐LDHs‐cGP embolization (NM‐LDHs 2 mg, cGAMP 40 µg). Under a surgical microscope, the abdominal aorta, hepatic artery, and gastro‐duodenal artery were carefully identified and dissected. Two ligatures were placed around the gastro‐duodenal artery, and the distal portion of the artery was ligated. Next, ligatures were placed around the abdominal aorta to temporarily interrupt blood flow to the arteries. Using a custom needle, a puncture was made upstream of the distal ligation point on the gastro‐duodenal artery, and a catheter was inserted into the hepatic artery. After the embolic microspheres were administered, the proximal portion of the gastro‐duodenal artery was ligated (upstream of the puncture point). Finally, the ligatures around the abdominal aorta were removed to restore blood flow to the hepatic artery.

For the combined treatment groups, when the implanted liver tumor reached a volume of 500–600 mm^3^, the rats were divided into the following groups: RFA (5 W, 10 s), RFA combined with NM‐LDHs embolization (2 mg), and RFA combined with NM‐LDHs‐cGP embolization (NM‐LDHs 2 mg, cGAMP 40 µg). Tumor size changes post‐treatment were monitored using ultrasound.

### 4.17 Immunohistochemistry (IHC) and HE Staining

Tumor tissues and vital organs were fixed in 4% paraformaldehyde solution for 24 h. The tissues were then dehydrated stepwise using ethanol and embedded in paraffin. Sections of 7–11 µm thickness were mounted on slides and dried in an incubator at 45°C. Paraffin was removed from the sections using xylene, followed by a series of ethanol washes, from high to low concentrations, and finally soaked in distilled water.

The sections were stained with hematoxylin and eosin (H&E) and observed under an optical microscope. For immunohistochemistry (IHC), primary antibodies targeting Ki‐67 were applied, followed by staining with a secondary antibody, aRbIgG (H+L)/HRP.

### 4.18 Immunofluorescence Staining and TUNEL

Tumor tissues were harvested from rats 14 days after different treatments and fixed in 4% paraformaldehyde for 48 h. After a series of processing steps, including tissue embedding, deparaffinization, antigen retrieval, blocking, incubation with primary antibodies (CD8 and Gzmb), incubation with secondary antibodies, staining, washing, periodic acid‐Schiff staining, and mounting, tumor tissue sections were observed under a fluorescence microscope (OLYMPUS, Japan).

After deparaffinization, tissue sections were washed three times with 1× PBST for 5 min each. Then, 20 µg/mL of proteinase K was added, and the sections were incubated at 37°C for 30 min. Subsequently, 50 µL of TUNEL reaction mixture (5 µL TdT enzyme + 45 µL fluorescent labeling solution + 50 µL TUNEL detection solution) was added to the tissue sections, which were incubated at 37°C in the dark for 60 min. Finally, DAPI was added, and the sections were incubated at room temperature for 3 min before imaging with a confocal microscope (OLYMPUS, Japan).

### 4.19 Biosafety Assays

To evaluate hemocompatibility, red blood cells were incubated with various concentrations of NM‐LDHs in a final volume of 1 mL. The mixture was then centrifuged, and the supernatant was discarded. After incubation, the mixture was centrifuged again, and the absorbance of the supernatant at 541 nm was measured using a microplate reader. The negative control group used an equivalent amount of red blood cells resuspended in saline, while the positive control group used deionized water. Hemolysis rate (HR) was calculated using the following formula:

$$\mathrm{HR}=\left(\mathrm{AS}-\mathrm{AN}\right)/\left(\mathrm{AP}-\mathrm{AN}\right)*100\%$$

In the formula, AS represents the absorbance value of the supernatant at 541 nm in the group treated with different concentrations of NM‐LDHs, AN represents the absorbance value of the supernatant at 541 nm in the saline group, and AP represents the absorbance value of the supernatant at 541 nm in the deionized water group.

For cytotoxicity evaluation, Hepa1–6, Huh7, and Thle2 cells were seeded into 96‐well plates at a density of 8 000 cells per well and incubated with different concentrations of LDHs for 24 h. Cell viability was assessed using the CCK‐8 kit.

After 14 days of RFA treatment, whole blood and serum were collected from mice and rats, and processed using an automatic hematology analyzer (Tekang, Jiangxi, China) and an automatic biochemical analyzer (Lanyun, Shenzhen, China), respectively. Vital organs were fixed and then stained with hematoxylin and eosin (H&E).

### 4.20 Capturation of BSA and TAAs by NM‐LDHs‐cGP

TAAs were prepared according to a previously described method. Fresh liver cancer tissues for RFA were ground in a cryogenic grinder. The resulting suspension was centrifuged at 5000 rpm for 5 min, and the supernatant was collected as TAAs. The prepared NM‐LDHs‐cGP (mass ratio 50:1) was then slowly added to solutions of BSA or TAAs at different mass ratios, vortexed for 2 min, and centrifuged at 20 000 g for 20 min. Unbound proteins in the supernatant were quantified using the BCA method and analyzed by SDS‐PAGE (10% Express Cast PAGE, NCM Biotech, Suzhou, China) kits. In the SDS‐PAGE experiment, 5 µg of TAAs were used as a positive control.

### 4.21 Generation of DCs and T Lymphocytes

DCs were prepared according to a previously described method. Mononuclear cells were isolated from the peripheral blood of healthy donors and cultured in DMEM medium containing 50 ng/mL GM‐CSF (PeproTech), 20 ng/mL IL‐4 (PeproTech), and 10% heat‐inactivated serum for 6 days. The medium and cytokines were replaced every 3 days, and cell differentiation was monitored under an optical microscope. The cells were then treated with cGAMP‐TAAs (cGAMP 2.5 µg, TAAs 12.5 µg), NM‐LDHs‐TAAs (NM‐LDHs 125 µg, TAAs 12.5 µg), or NM‐LDHs‐cGP‐TAAs (NM‐LDHs 125 µg, cGAMP 2.5 µg, TAAs 12.5 µg) for 24 h. The collect cells were stained with various antibodies and dected by flow cytometry. Naïve T cells were isolated from PBMCs using a human naïve CD8+ T cell isolation kit (Stemcell Technologies, 19258) and incubated with antigen‐specific DCs at a 5:2 ration in RPMI 1640 medium with 25 U mL^−1^ IL‐2 (PeproTech, 200–02). The expanded cell numbers were quantified using a cell counter (RWD, C100‐SE). The collect cells were stained with various antibodies and dected by flow cytometry. The date was analyzed with FlowJo software.

### 4.22 Cytotoxicity Assays

GFP‐tagged human hepatocellular carcinoma cell line Huh‐7 were employed and co‐cultured GFP+ Huh‐7 cells with differently treated CD8+ T cells at 37°C for 18 h at an effector‐to‐target (E/T) cell ratio of 10:1. The cytotoxic activity of CD8+ T cells was dynamically monitored using a high‐content imaging and analysis system (Molecular Devices, ImageXpress Micro Confocal). Additionally, the changes in green fluorescence intensity at the bottom layer of the culture were observed under an inverted fluorescence microscope (Olympus, IX73) to assess the cytotoxic effects. Furthermore, lactate dehydrogenase (LDHA) activity was measured using an LDHA Cytotoxicity Assay Kit (Beyotime, C0016) to quantify cell lysis. To determine maximum LDH release, cells were treated with 10% Triton X‐100, and absorbance was measured at 490 nm using a microplate reader (Biotek, CYTATION 1). The percentage of LDH cytotoxicity was calculated according to the manufacturer's formula: LDH cytotoxicity (%)  =  ((experimental value A490) – (spontaneous release A490))/ ((maximum release A490) – (spontaneous release A490)) × 100.

### 4.23 Cytokine Detection

For analysis of the cytokines of CD8+ T cell, 5 days after treatment, the supernatant after centrifugation was collected for further analysis. The cytokine levels of IFN‐γ and TNF‐α were measured with ELISA kits (Thermofisher Scientific, 88‐7346‐88 and Servicebio, GEH0006‐1) according to the manufacturer's instructions.

### 4.24 Statistical Analysis

Data were analyzed using a two‐tailed Student's t‐test or analysis of variance (ANOVA) with GraphPad Prism 10 software (https://graphpad.com/scientific‐software/prism), and results are expressed as mean ± standard error of the mean (SEM). Overall survival analysis was performed using the Kaplan‐Meier method, and statistical analysis was conducted with the log‐rank test (Mantel‐Cox). Statistical significance was set at ^*^ *P* < 0.05, ^**^ *P* < 0.01, ^***^ *P* < 0.001, ^****^ *P* < 0.0001, with ns indicating no significance.

## Author Contributions

H.Q., D.J, T.Z., L. G., Z. L., and C.W. conceived and designed the study. H.Q., D.J, T.Z., Z.C., Z.S., Z.W., Z.T., G.Z., and S.Z. performed the experiments. H.Q., D. J, C.W., T.Z., and Z. L. participated in data analysis. The manuscript was written by H.Q. and C.W.

## Conflicts of Interest

The authors declare no conflicts of interest.

## Supporting information




**Supporting File 1**: advs74907‐sup‐0001‐FiguresS1.pdf.

**Supporting File 2**: advs74907‐sup‐0002‐Table1.pdf.

## Data Availability

The data that support the findings of this study are available from the corresponding author upon reasonable request.

## References

[1] N. Ganne‐Carrie and P. Nahon , “Differences between Hepatocellular Carcinoma Caused by Alcohol and Other Aetiologies,” Journal of Hepatology 82, no. 5 (2025): 909–917. 10.1016/j.jhep.2024.12.030 39710147

[2] T. Tong , W. Gao , H. Jian , et al., “The Role and Potential Mechanisms of Exosomes in the Progression of Hepatocellular Carcinoma,” Holistic Integrative Oncology 4, no. 1 (2025): 36.

[3] European Association for the Study of the Liver , “EASL Clinical Practice Guidelines: Management of Hepatocellular Carcinoma,” Journal of Hepatology 69, no. 1 (2018): 182–236. 10.1016/j.jhep.2018.03.019 29628281

[4] European Association for the Study of the Liver , “EASL 2017 EASL 2017 Clinical Practice Guidelines on the management of hepatitis B virus infection,” Journal of Hepatology 67, no. 2 (2017): 370–398. 10.1016/j.jhep.2017.03.021 28427875

[5] T. Taniai , S. Shimada , Y. Akiyama , et al., “Integrative Transcriptome Profiling Elucidates Molecular and Immunovascular Characteristics of Macrotrabecular Hepatocellular Carcinoma,” Hepatology 83 (2025): 231–248. 10.1097/HEP.0000000000001284 PMC12799264 40009602

[6] A. G. Singal , F. Kanwal , and J. M. Llovet , “Global Trends in Hepatocellular Carcinoma Epidemiology: Implications for Screening, Prevention and Therapy,” Nature Reviews Clinical Oncology 20, no. 12 (2023): 864–884. 10.1038/s41571-023-00825-3 37884736

[7] J. M. Llovet , T. De Baere , L. Kulik , et al., “Locoregional Therapies in the Era of Molecular and Immune Treatments for Hepatocellular Carcinoma,” Nature Reviews Gastroenterology & Hepatology 18, no. 5 (2021): 293–313. 10.1038/s41575-020-00395-0 33510460

[8] M. Xi , Z. Yang , L. Hu , et al., “Radiofrequency Ablation versus Stereotactic Body Radiotherapy for Recurrent Small Hepatocellular Carcinoma: A Randomized, Open‐Label, Controlled Trial,” Journal of Clinical Oncology 43, no. 9 (2025): 1073–1082. 10.1200/JCO-24-01532 39693584

[9] Y.‐J. Zhang , J. Chen , Z. Zhou , et al., “Transarterial Chemoembolization with Radiofrequency Ablation versus Surgical Resection for Small Late‐Recurrence Hepatocellular Carcinoma,” Radiology 314, no. 2 (2025): 241096. 10.1148/radiol.241096 39903071

[10] L. Wang , L. Zhu , C. Liang , et al., “Targeting N6‐methyladenosine Reader YTHDF1 with siRNA Boosts Antitumor Immunity in NASH‐HCC by Inhibiting EZH2‐IL‐6 Axis,” Journal of Hepatology 79, no. 5 (2023): 1185–1200. 10.1016/j.jhep.2023.06.021 37459919

[11] X. Sun , Z. Yang , J. Mei , et al., “The Guiding Value of Microvascular Invasion for Treating Early Recurrent Small Hepatocellular Carcinoma,” International Journal of Hyperthermia 38, no. 1 (2021): 931–938. 10.1080/02656736.2021.1937715 34121576

[12] W. Sieghart , F. Hucke , and M. Peck‐Radosavljevic , “Transarterial Chemoembolization: Modalities, Indication, and Patient Selection,” Journal of Hepatology 62, no. 5 (2015): 1187–1195. 10.1016/j.jhep.2015.02.010 25681552

[13] D. Wang and W. Rao , “Bench‐to‐bedside Development of Multifunctional Flexible Embolic Agents,” Theranostics 13, no. 7 (2023): 2114–2139. 10.7150/thno.80213 PMC10157739 37153738

[14] Y. Yuan , W. He , Z. Yang , et al., “TACE‐HAIC Combined with Targeted Therapy and Immunotherapy versus TACE Alone for Hepatocellular Carcinoma with Portal Vein Tumour Thrombus: A Propensity Score Matching Study,” International Journal of Surgery 109, no. 5 (2023): 1222–1230. 10.1097/JS9.0000000000000256 PMC10389515 37026861

[15] X. Chen , X. Wu , W. Peng , et al., “Combined TACE with Targeted and Immunotherapy versus TACE Alone Improves DFS in HCC with MVI: A Multicenter Propensity Score Matching Study,” Journal of Hepatocellular Carcinoma 12 (2025): 561–577. 10.2147/JHC.S504016 PMC11930282 40124969

[16] C. Wei , C. Wu , X. Jin , et al., “CT/MR Detectable Magnetic Microspheres for Self‐regulating Temperature Hyperthermia and Transcatheter Arterial Chemoembolization,” Acta Biomaterialia 153 (2022): 453–464. 10.1016/j.actbio.2022.09.054 36167241

[17] L. Li , Y. Cao , H. Zhang , et al., “Temperature Sensitive Nanogel‐stabilized Pickering Emulsion of Fluoroalkane for Ultrasound Guiding Vascular Embolization Therapy,” Journal of Nanobiotechnology 21, no. 1 (2023): 413. 10.1186/s12951-023-02181-x PMC10634024 37946199

[18] H. Choi , B. Choi , B. Yu , et al., “On‐demand Degradable Embolic Microspheres for Immediate Restoration of Blood Flow during Image‐guided Embolization Procedures,” Biomaterials 265 (2021): 120408. 10.1016/j.biomaterials.2020.120408 PMC7673262 32992115

[19] C. Wang , L. Zhong , J. Xu , et al., “Oncolytic Mineralized Bacteria as Potent Locally Administered Immunotherapeutics,” Nature Biomedical Engineering 8, no. 5 (2024): 561–578. 10.1038/s41551-024-01191-w 38514774

[20] T. Hu , Z. Gu , G. R. Williams , et al., “Layered Double Hydroxide‐based Nanomaterials for Biomedical Applications,” Chemical Society Reviews 51, no. 14 (2022): 6126–6176. 10.1039/d2cs00236a 35792076

[21] M. Chang , M. Wang , B. Liu , et al., “A Cancer Nanovaccine Based on an FeAl‐Layered Double Hydroxide Framework for Reactive Oxygen Species‐Augmented Metalloimmunotherapy,” ACS Nano 18, no. 11 (2024): 8143–8156. 10.1021/acsnano.3c11960 38436248

[22] Q. Hu , H. Zuo , J. C. Hsu , et al., “The Emerging Landscape for Combating Resistance Associated with Energy‐Based Therapies via Nanomedicine,” Advanced Materials 36, no. 5 (2024): 2308286. 10.1002/adma.202308286 PMC10872442 37971203

[23] W. Tang , J. Wu , L. Wang , et al., “Bioactive Layered Double Hydroxides for Synergistic Sonodynamic/Cuproptosis Anticancer Therapy with Elicitation of the Immune Response,” ACS Nano 18, no. 15 (2024): 10495–10508. 10.1021/acsnano.3c11818 38556991

[24] Y. Yang , T. Hu , K. Zhao , et al., “Metal Doping Enabling Defective CoMo‐Layered Double Hydroxide Nanosheets as Highly Efficient Photosensitizers for NIR‐II Photodynamic Cancer Therapy,” Advanced Materials 37, no. 4 (2025): 2405847. 10.1002/adma.202405847 39629533

[25] A. Vitale , P. Romano , U. Cillo , et al., “Liver Resection vs Nonsurgical Treatments for Patients with Early Multinodular Hepatocellular Carcinoma,” JAMA Surgery 159, no. 8 (2024): 881–889. 10.1001/jamasurg.2024.1184 PMC11097094 38771633

[26] R. LIAO , P. SONG , Y. DUAN , et al., “A Well‐matched Marriage of Immunotherapy and Radiofrequency Ablation to Reduce the Relapse and Progression of Hepatocellular Carcinoma,” Biosci Trends 16, no. 5 (2022): 377–380. 10.5582/bst.2022.01373 36089338

[27] Z. Li , Z. Chen , Y. Gao , et al., “Shape Memory Micro‐anchors with Magnetic Guidance for Precision Micro‐vascular Deployment,” Biomaterials 283 (2022): 121426. 10.1016/j.biomaterials.2022.121426 35240471

[28] E. Griswold , J. Cappello , and H. Ghandehari , “Silk‐elastinlike Protein‐based Hydrogels for Drug Delivery and Embolization,” Advanced Drug Delivery Reviews 191 (2022): 114579. 10.1016/j.addr.2022.114579 36306893

[29] G. Li , L. Ye , J. Pan , et al., “Antitumoural Hydroxyapatite Nanoparticles‐Mediated Hepatoma‐Targeted Trans‐Arterial Embolization Gene Therapy: In Vitro and in Vivo Studies,” Liver International 32, no. 6 (2012): 998–1007. 10.1111/j.1478-3231.2012.02761.x 22340582

[30] G. Jia , J. Van Valkenburgh , A. Z. Chen , et al., “Recent Advances and Applications of Microspheres and Nanoparticles in Transarterial Chemoembolization for Hepatocellular Carcinoma,” WIREs Nanomedicine and Nanobiotechnology 14, no. 2 (2022): 1749. 10.1002/wnan.1749 PMC8850537 34405552

[31] G. Yuan , Z. Liu , W. Wang , et al., “Multifunctional Nanoplatforms Application in the Transcatheter Chemoembolization against Hepatocellular Carcinoma,” Journal of Nanobiotechnology 21, no. 1 (2023): 68. 10.1186/s12951-023-01820-7 PMC9969656 36849981

[32] X.‐M. Bai , M. Cui , W. Yang , et al., “The 10‐year Survival Analysis of Radiofrequency Ablation for Solitary Hepatocellular Carcinoma 5 cm or Smaller: Primary versus Recurrent HCC,” Radiology 300, no. 2 (2021): 458–469. 10.1148/radiol.2021200153 34003058

[33] K. K. Ng , R. T. Poon , C. M. Lam , J. Yuen , W. K. Tso , and S. T. Fan , “Efficacy and Safety of Radiofrequency Ablation for Perivascular Hepatocellular Carcinoma without Hepatic Inflow Occlusion,” British Journal of Surgery 93, no. 4 (2006): 440–447. 10.1002/bjs.5267 16470712

[34] Y. Zhao , T. Yang , Y. Ouyang , et al., “Radiofrequency Ablation Plays Double Role in Immunosuppression and Activation of PBMCs in Recurrent Hepatocellular Carcinoma,” Frontiers in Immunology 15 (2024): 1339213. 10.3389/fimmu.2024.1339213 PMC10859425 38348038

[35] Y. Jin , Q. Zhao , C. Fan , et al., “Peripheral T‐Cell Subsets in Radiofrequency Ablation for Tumors from Different Origins,” Asian Journal of Surgery 47, no. 3 (2024): 1378–1382. 10.1016/j.asjsur.2023.12.089 38160147

[36] X. Zhang , B. Yang , Q. Ni , and X. Chen , “Materials Engineering Strategies for Cancer Vaccine Adjuvant Development,” Chemical Society Reviews 52, no. 9 (2023): 2886–2910. 10.1039/d2cs00647b 37014050

[37] T. Zhao , Y. Cai , Y. Jiang , et al., “Vaccine Adjuvants: Mechanisms and Platforms,” Signal Transduction and Targeted Therapy 8, no. 1 (2023): 283. 10.1038/s41392-023-01557-7 PMC10356842 37468460

[38] Z. Su , H. Boucetta , J. Shao , et al., “Next‐generation Aluminum Adjuvants: Immunomodulatory Layered Double Hydroxide NanoAlum Reengineered from First‐line Drugs,” Acta Pharmaceutica Sinica B 14, no. 11 (2024): 4665–4682. 10.1016/j.apsb.2024.09.012 PMC11628803 39664431

[39] X. Zhang , X. C. Bai , and Z. J. Chen , “Structures and Mechanisms in the cGAS‐STING Innate Immunity Pathway,” Immunity 53, no. 1 (2020): 43–53. 10.1016/j.immuni.2020.05.013 32668227

[40] L. Li , I. Soyhan , E. Warszawik , and P. van Rijn , “Layered Double Hydroxides: Recent Progress and Promising Perspectives toward Biomedical Applications,” Advanced Science 11, no. 20 (2024): 2306035. 10.1002/advs.202306035 PMC11132086 38501901

[41] W. Fan , B. Zhu , S. Chen , et al., “Survival in Patients with Recurrent Intermediate‐Stage Hepatocellular Carcinoma,” JAMA Oncology 10, no. 8 (2024): 1047–1054. 10.1001/jamaoncol.2024.1831 PMC11190833 38900435

[42] K. R. Patel , H. Menon , R. R. Patel , E. P. Huang , V. Verma , and F. E. Escorcia , “Locoregional Therapies for Hepatocellular Carcinoma,” JAMA Network Open 7, no. 11 (2024): 2447995. 10.1001/jamanetworkopen.2024.47995 PMC12527482 39602117

[43] Y. J. Zhang , M. S. Chen , Y. Chen , W. Y. Lau , and Z. Peng , “Long‐Term Outcomes of Transcatheter Arterial Chemoembolization Combined with Radiofrequency Ablation as an Initial Treatment for Early‐Stage Hepatocellular Carcinoma,” JAMA Network Open 4, no. 9 (2021): 2126992. 10.1001/jamanetworkopen.2021.26992 PMC8477266 34570206

[44] L. Li , H. S. Zhang , H. Zhao , et al., “Radiofrequency‐thermal Effect of Cisplatin‐Crosslinked Nanogels for Triple Therapies of Ablation‐Chemo‐Embolization ,” Chemical Engineering Journal 450 (2022): 138421.

[45] C. F. Gonsalves and D. B. Brown , “Chemoembolization of Hepatic Malignancy,” Abdominal Imaging 34, no. 5 (2009): 557–565. 10.1007/s00261-008-9446-y 18668189

[46] J. E. Song and D. Y. Kim , “Conventional vs Drug‐Eluting Beads Transarterial Chemoembolization for Hepatocellular Carcinoma,” World Journal of Hepatology 9, no. 18 (2017): 808–814. 10.4254/wjh.v9.i18.808 PMC5491403 28706579

[47] R. S. Riley , C. H. June , R. Langer , and M. J. Mitchell , “Delivery Technologies for Cancer Immunotherapy,” Nature Reviews Drug Discovery 18, no. 3 (2019): 175–196. 10.1038/s41573-018-0006-z PMC6410566 30622344

[48] N. Chong , L. Yao , M. Wang , and Y. Xu , “The Evolving Landscape of Bench‐to‐bedside Translation in the Era of Neoadjuvant Platforms: Perspectives and Opportunities,” Holistic Integrative Oncology 5, no. 1 (2026): 13.

