# Supplementary material for: Smartly Assembled near‐Micron RF‐Responsive Agents Bridge Transarterial Embolization and Immunothermal Potentiation
Source: Adv Sci (Weinh). 2026 Mar 26;13(31):e00067. doi: 10.1002/advs.202600067 (PMC13587982; doi:10.1002/advs.202600067)
Supplement: Supplementary file 1 [file ADVS-13-e00067-s001.pdf]

Extended data Table

Smartly Assembled Near-Micron RF-Responsive Agents Bridge Transarterial Embolization and Immunothermal Potentiation

Qitao Hu[1#], Junkang Ding[1#], Lingxiao Zhang[8#], Zhou Tian[1], Cheng Zeng[1], ShaoMing Zou[1], Wenjing Zhang[1], Tangye Zeng[1], Zhencang Gao[1], Zhouyi Sun[1], Gang Liu[5,6, 7], Weiyu Chen[2,3*], Zhe Tang[1,4*]*

1 Department of Surgery, the Fourth Affiliated Hospital of School of Medicine, and International School of Medicine, International Institutes of Medicine, Zhejiang University, Yiwu, China, 322000

2 Department of Respiratory and Critical Care Medicine, Center for Oncology Medicine, the Fourth Affiliated Hospital of School of Medicine, and International School of Medicine, International Institutes of Medicine, Zhejiang University, Yiwu, China, 322000

3 Zhejiang Key Laboratory of Precision Diagnosis and Treatment for Lung Cancer, Yiwu, China, 322000

4 Department of Surgery, The Second Affiliated Hospital, Zhejiang University School of Medicine, Hangzhou, China

5 State Key Laboratory of Molecular Vaccinology and Molecular Diagnostics, National Institute of Diagnostics and Vaccine Development in Infectious Diseases, Center for Molecular Imaging and Translational Medicine, School of Public Health, Xiamen University, Xiamen, China.

6 State Key Laboratory of Cellular Stress Biology, Innovation Center for Cell Biology, School of Life Sciences, Xiamen University, Xiamen, China

7 MOE Key Laboratory of Spectrochemical Analysis & Instrumentation, College of Chemistry and Chemical Engineering, Xiamen University, Xiamen, China.

8 Interdisciplinary Nanoscience Center, Aarhus University, Aarhus C 8000, Denmark.

*Correspondence:

Prof. Gang Liu Email: gangliu.cmitm@xmu.edu.cn

Prof. Weiyu Chen Email: weiyuchen@zju.edu.cn

Prof. Zhe Tang Email: 8xi@zju.edu.cn

#Dr. Qitao Hu, Dr. Junkang Ding and Dr. Lingxiao Zhang equally contributed to the work.

Lead Contact: Prof. Zhe Tang

Table 1. Baseline characteristics of the patient

No.	Preoperative tumor diameter (cm)	Postoperative Recurrence (month)
1	2.3	none
2	1.5, 1.2	6
3	1.2	none
4	2.5	none
5	2.0	2
6	1.7	none
7	2.6, 0.8	2
8	2.6	none
9	1.4	none
10	2.6	none
11	1.1	none
12	2.5	none
13	2.8	2
14	2.0	none
15	1.0	none
16	1.3	none
17	2.3	none
18	1.0	12
19	1.5	none
20	1.7, 1.6	2
21	2.1	12
22	4.0	12
23	2.2	none
24	3.3	none
25	1.2	12

26	2.2	7
27	1.4	2
28	1.8	4
29	1.3	none
30	2.0	1
31	1.9	none
32	2.2	none
33	1.0	none
34	1.8，1.2	none
35	1.9	none
36	2.8	6
37	2.1	5
38	1.2	none
39	1.5	none
40	2.0	none
41	1.5	none
42	2.0	none
43	3.0	5
44	1.5	none
45	1.0	2
46	2.5	8
47	3.0	none
48	2.5	none
49	1.8	none
50	1.8	7
51	1.5	none
52	1.6	none
53	0.9	none
54	1.6	none
55	1.6	none
56	1.8	12
57	1.4	none
58	1.2	none
59	1.0	none
