# Supplementary material for: Smartly Assembled near‐Micron RF‐Responsive Agents Bridge Transarterial Embolization and Immunothermal Potentiation
Source: Adv Sci (Weinh). 2026 Mar 26;13(31):e00067. doi: 10.1002/advs.202600067 (PMC13587982; doi:10.1002/advs.202600067)
Supplement: Supplementary file 2 [file ADVS-13-e00067-s002.pdf]

Extended data Figure

Smartly Assembled Near-Micron RF-Responsive Agents Bridge Transarterial Embolization and Immunothermal Potentiation

Qitao Hu[1#], Junkang Ding[1#], Lingxiao Zhang[8#], Zhou Tian[1], Cheng Zeng[1], ShaoMing Zou[1], Wenjing Zhang[1], Tangye Zeng[1], Zhencang Gao[1], Zhouyi Sun[1], Gang Liu[5,6, 7], Weiyu Chen[2,3*], Zhe Tang[1,4*]*

1 Department of Surgery, the Fourth Affiliated Hospital of School of Medicine, and International School of Medicine, International Institutes of Medicine, Zhejiang University, Yiwu, China, 322000

2 Department of Respiratory and Critical Care Medicine, Center for Oncology Medicine, the Fourth Affiliated Hospital of School of Medicine, and International School of Medicine, International Institutes of Medicine, Zhejiang University, Yiwu, China, 322000

3 Zhejiang Key Laboratory of Precision Diagnosis and Treatment for Lung Cancer, Yiwu, China, 322000

4 Department of Surgery, The Second Affiliated Hospital, Zhejiang University School of Medicine, Hangzhou, China

5 State Key Laboratory of Molecular Vaccinology and Molecular Diagnostics, National Institute of Diagnostics and Vaccine Development in Infectious Diseases, Center for Molecular Imaging and Translational Medicine, School of Public Health, Xiamen University, Xiamen, China.

6 State Key Laboratory of Cellular Stress Biology, Innovation Center for Cell Biology, School of Life Sciences, Xiamen University, Xiamen, China

7 MOE Key Laboratory of Spectrochemical Analysis & Instrumentation, College of Chemistry and Chemical Engineering, Xiamen University, Xiamen, China.

8 Interdisciplinary Nanoscience Center, Aarhus University, Aarhus C 8000, Denmark.

*Correspondence:

Prof. Gang Liu Email: gangliu.cmitm@xmu.edu.cn

Prof. Weiyu Chen Email: weiyuchen@zju.edu.cn

Prof. Zhe Tang Email: 8xi@zju.edu.cn

#Dr. Qitao Hu, Dr. Junkang Ding and Dr. Lingxiao Zhang equally contributed to the work.

Lead Contact: Prof. Zhe Tang

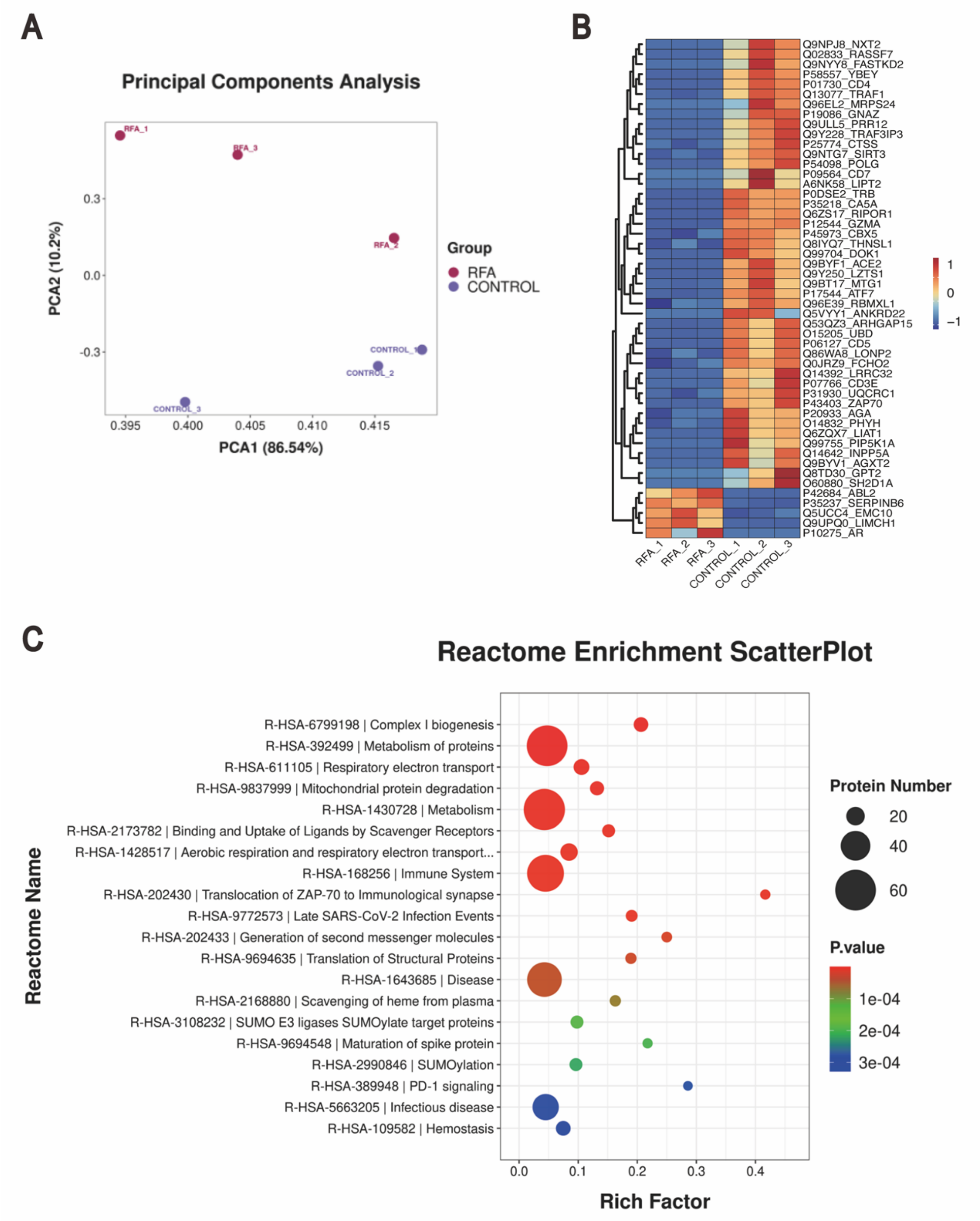

Supplementary Fig.1 A, Principal component analysis (PCA) of protein expression profiles. B, Heatmap showing the top 50 differentially expressed genes between the RFA group and Surgical resection group. C**,** Reactome Enrichment analysis of the proteomics datasets.

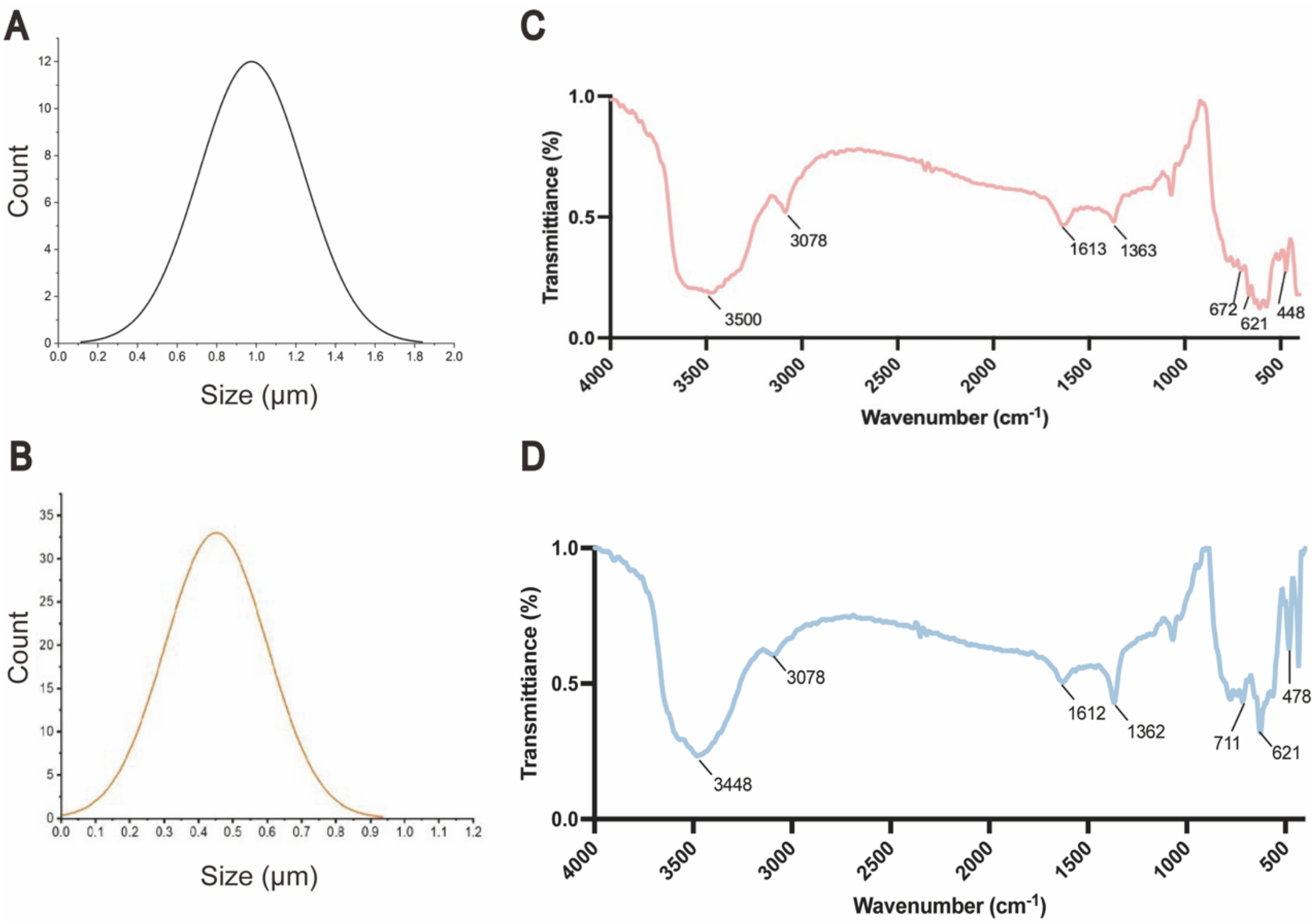

Supplementary Fig.2 A, Particle size distribution of NM-LDHs. B, Particle size distribution of m-LDHs. C, Fourier transform infrared (FTIR) spectroscopy of NM-LDHs. D, Fourier transform infrared (FTIR) spectroscopy of m-LDHs.

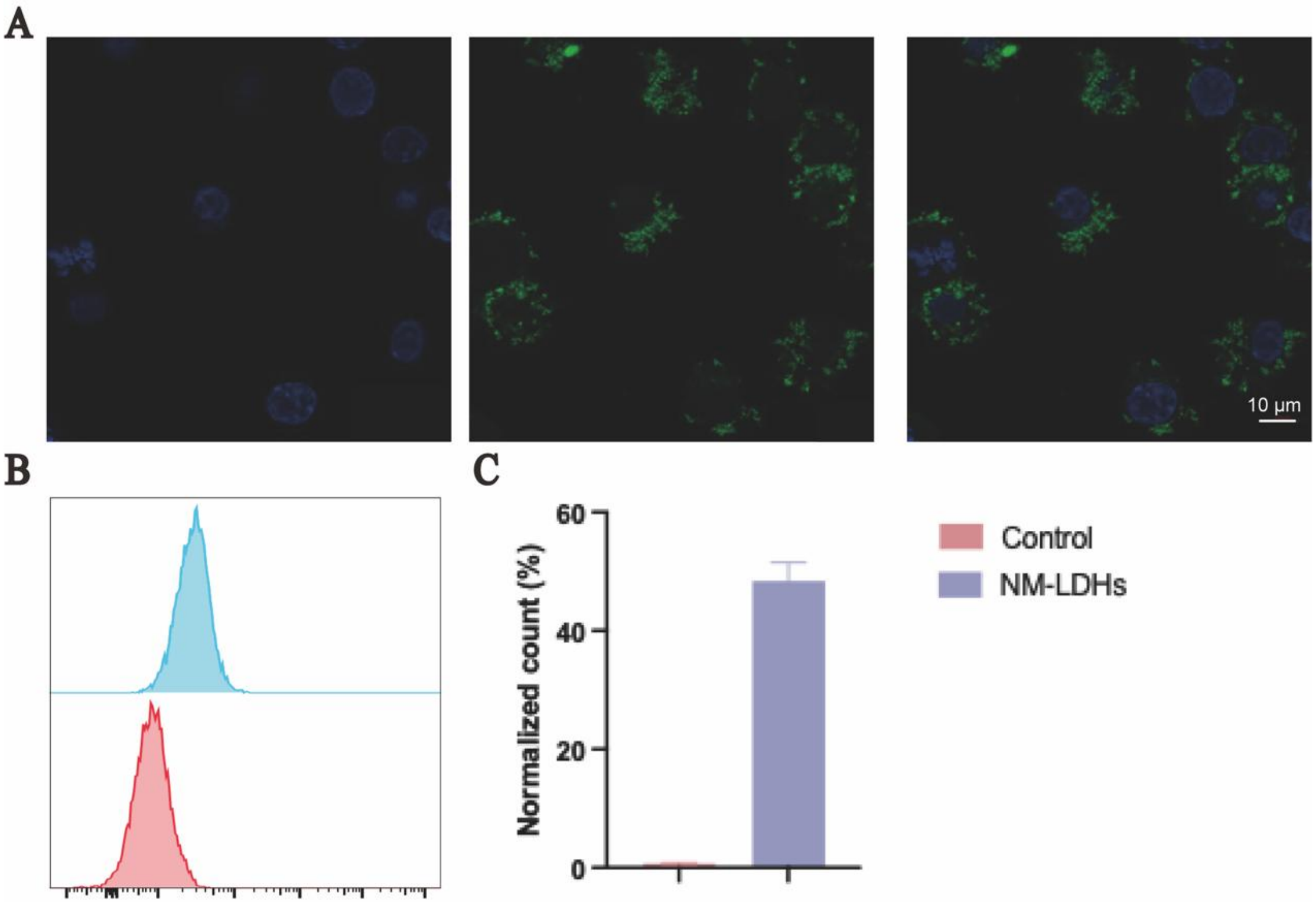

Supplementary Fig.3 A-B Internalization of NM-LDHs-FITC by Hepa1-6 cells observed via

confocal microscopy (A) and flow cytometry (B-C). Scale bar: 10 μm.

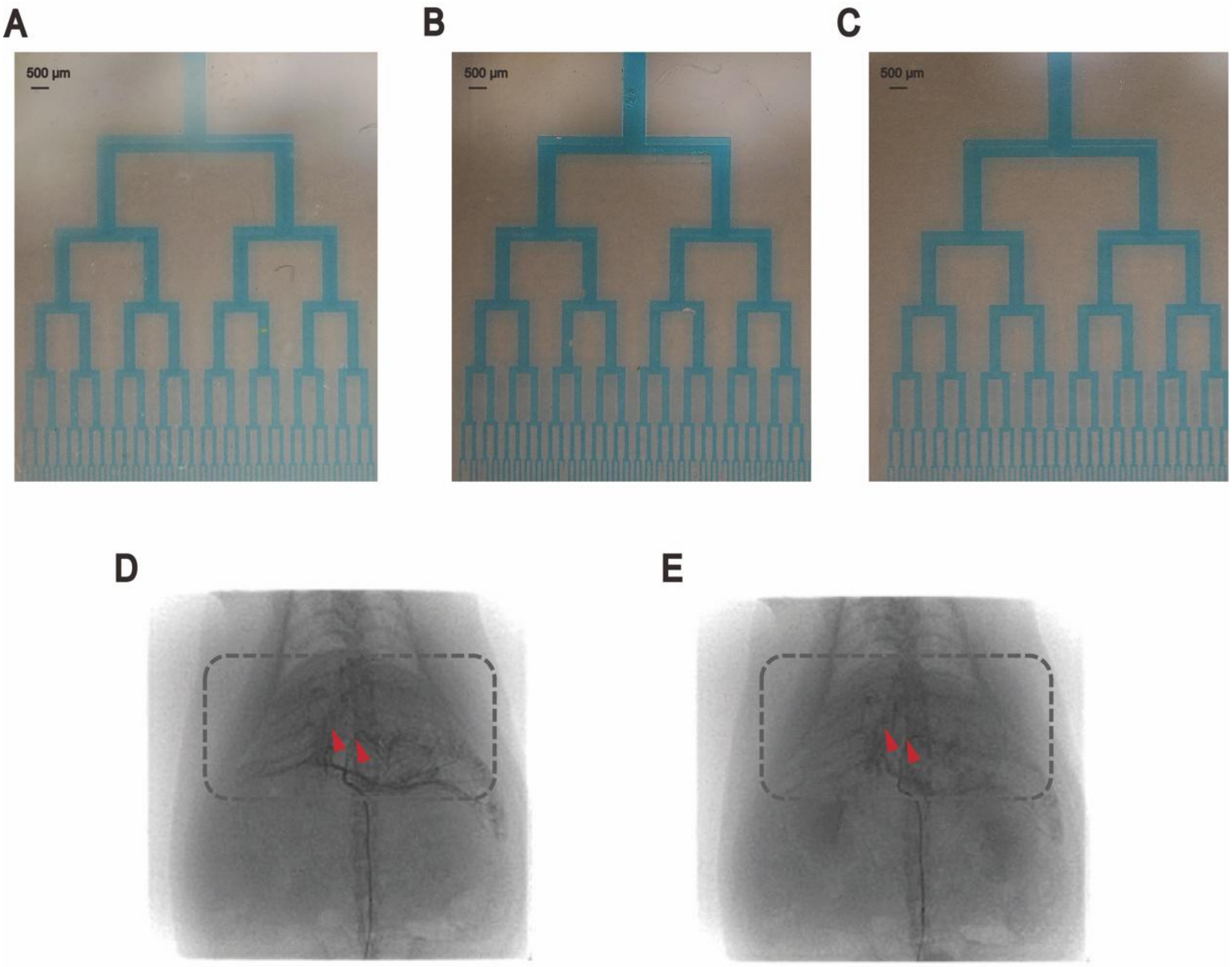

Supplementary Fig.4 A-C, Verification of microfluidic chip (A: m-LDHs, B: NM-LDHs, C:Lipiodol) patency by red ink injection Scale bar: 500 μm**.** D-E, X-ray fluoroscopy images of rat hepatic arteries before (D) and after (E) embolization with s-LDHs.

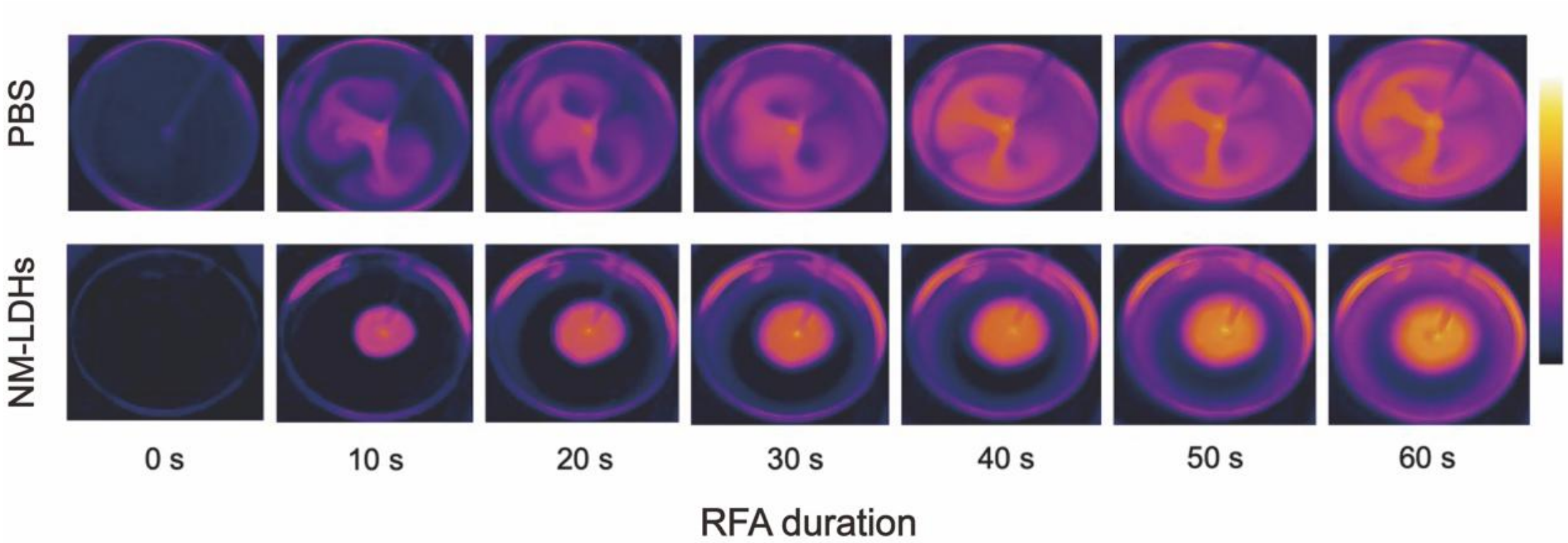

Supplementary Fig.5 A, Infrared thermal imaging of PBS and NM-LDHs groups under 5 W radiofrequency power in Vitro.

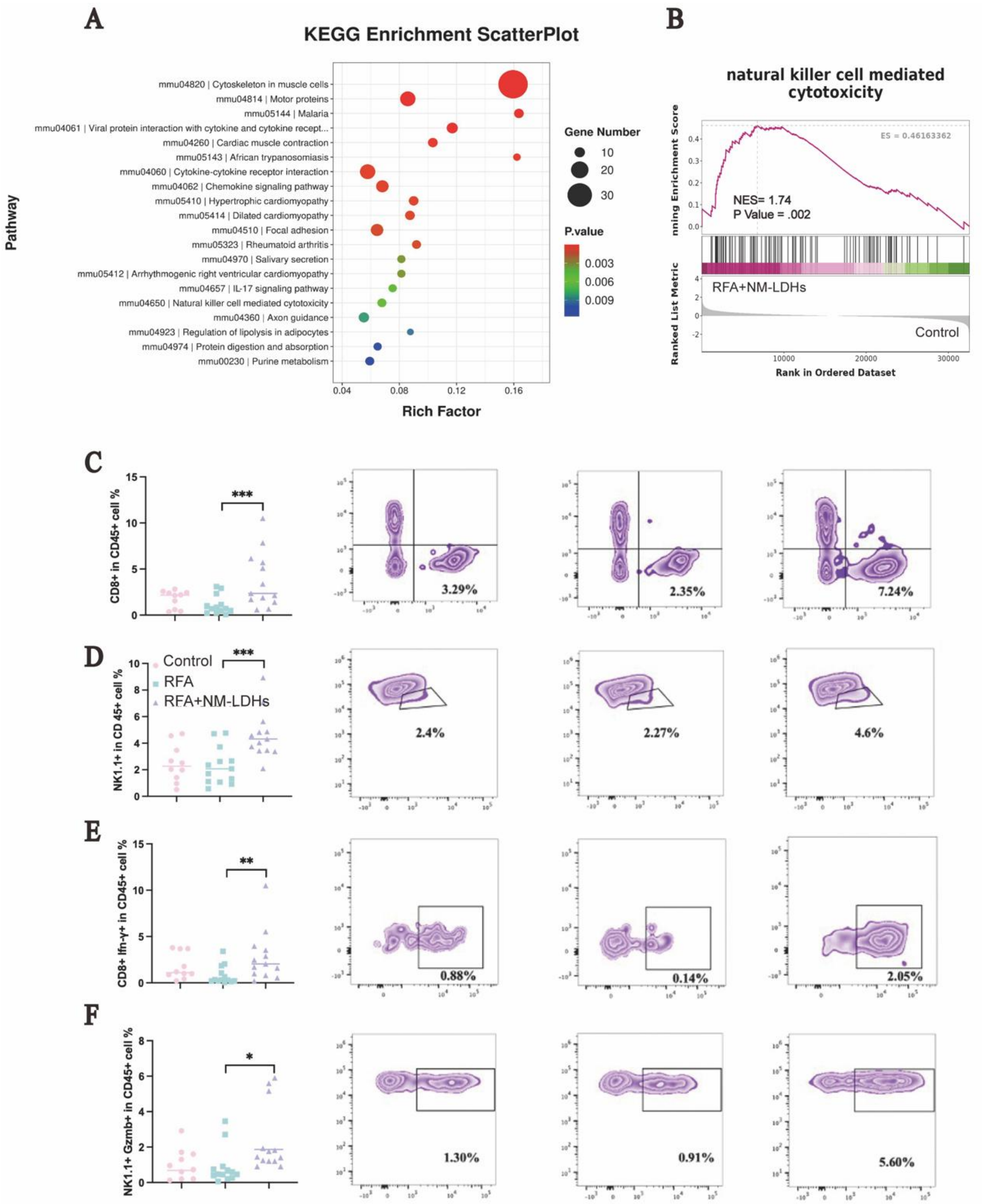

Supplementary Fig.6 A, KEGG enrichment analysis of the transcriptomics datasets. B, GSEA analysis of pathway activity. The curves represent changes in the enrichment score (NES). P-values and FDR values indicate statistical significance. C-D, Quantification results of the percentage of CD8+ T cells (C) and NK cells (D) in tumors at 14 days post-treatment (n = 13). E-F: Quantitative analysis of the proportion of Ifnγ-positive CD8+ T cells (E) and Granzyme B (GZMB)-positive NK cells (F) in tumors at 14 days post-treatment. Data presented as mean ± s.d. All replicates are biological. One-way ANOVA was performed for statistical significance. **P* < 0.05, ***P* < 0.01, ****P* < 0.001, *****P* < 0.0001, ns, not statistical significance.

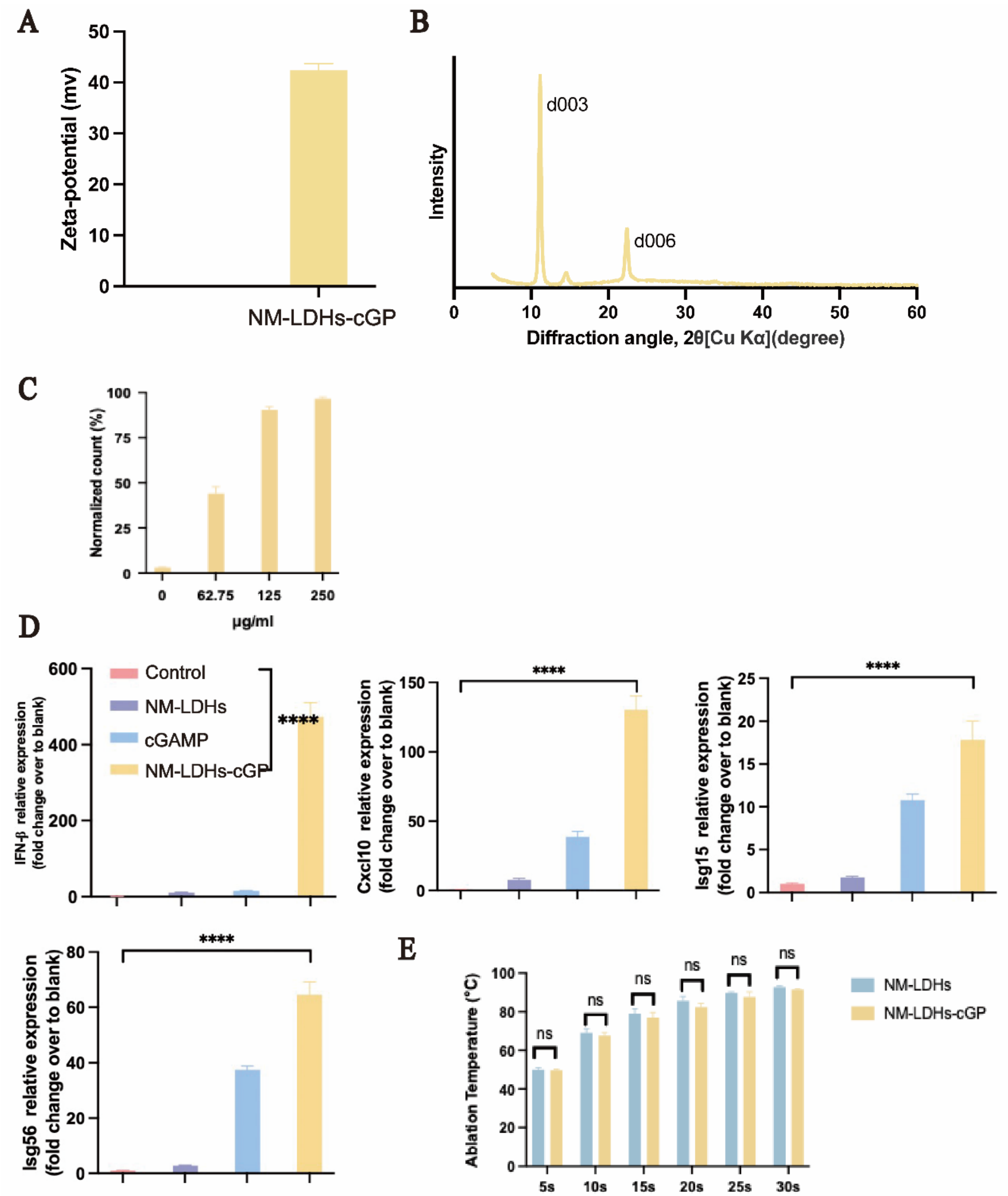

Supplementary Fig.7 A, Zeta potential analysis of NM-LDHs-cGP. B, XRD patterns of NM-LDHs-cGP. C. Cellular uptake efficiency of NM-LDHs-cGP by DC2.4 cells at different concentrations D, qPCR analysis of STING pathway activation in Hepa1-6 cells. E, Comparison of intratumoral heating effects between NM-LDHs and NM-LDHs-cGP. Data presented as mean ± s.d. All replicates are biological. Two-tailed Student's *t*-test was performed for statistical significance. *$P < 0.05$, **$P < 0.01$, ***$P < 0.001$, ****$P < 0.0001$, ns, not statistical significance.

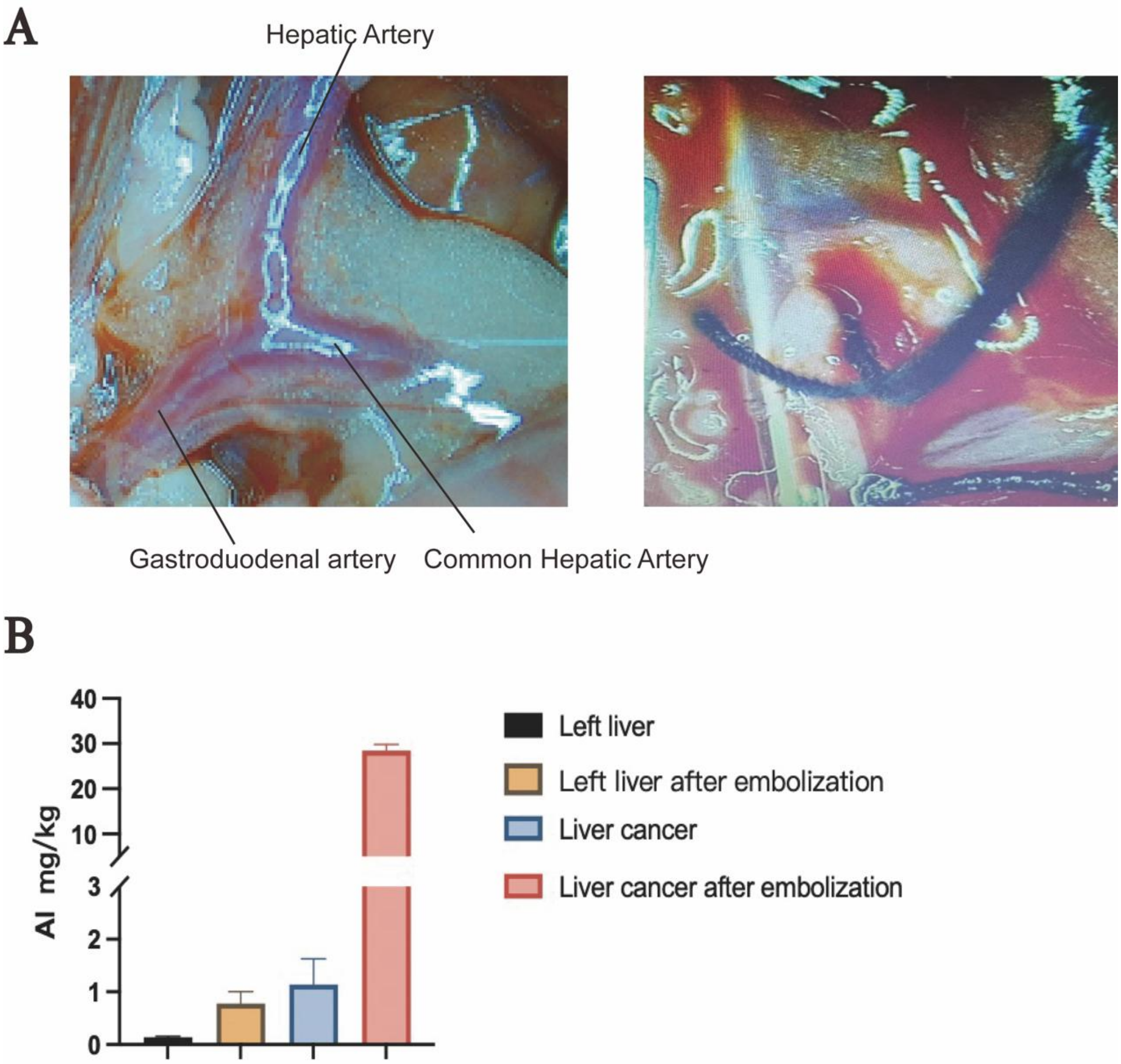

Supplementary Fig.8 A, Retrograde catheterization of the hepatic artery. B, Inductively Coupled Plasma-Optical Emission Spectrometry (ICP-OES) detection of Aluminum in the left liver and tumor before and after embolization. C, Complete blood count and blood biochemical analysis at 14 days post-treatment. E, Histopathological changes in major organs (heart, liver, spleen, lung, and kidney) across different treatment groups.

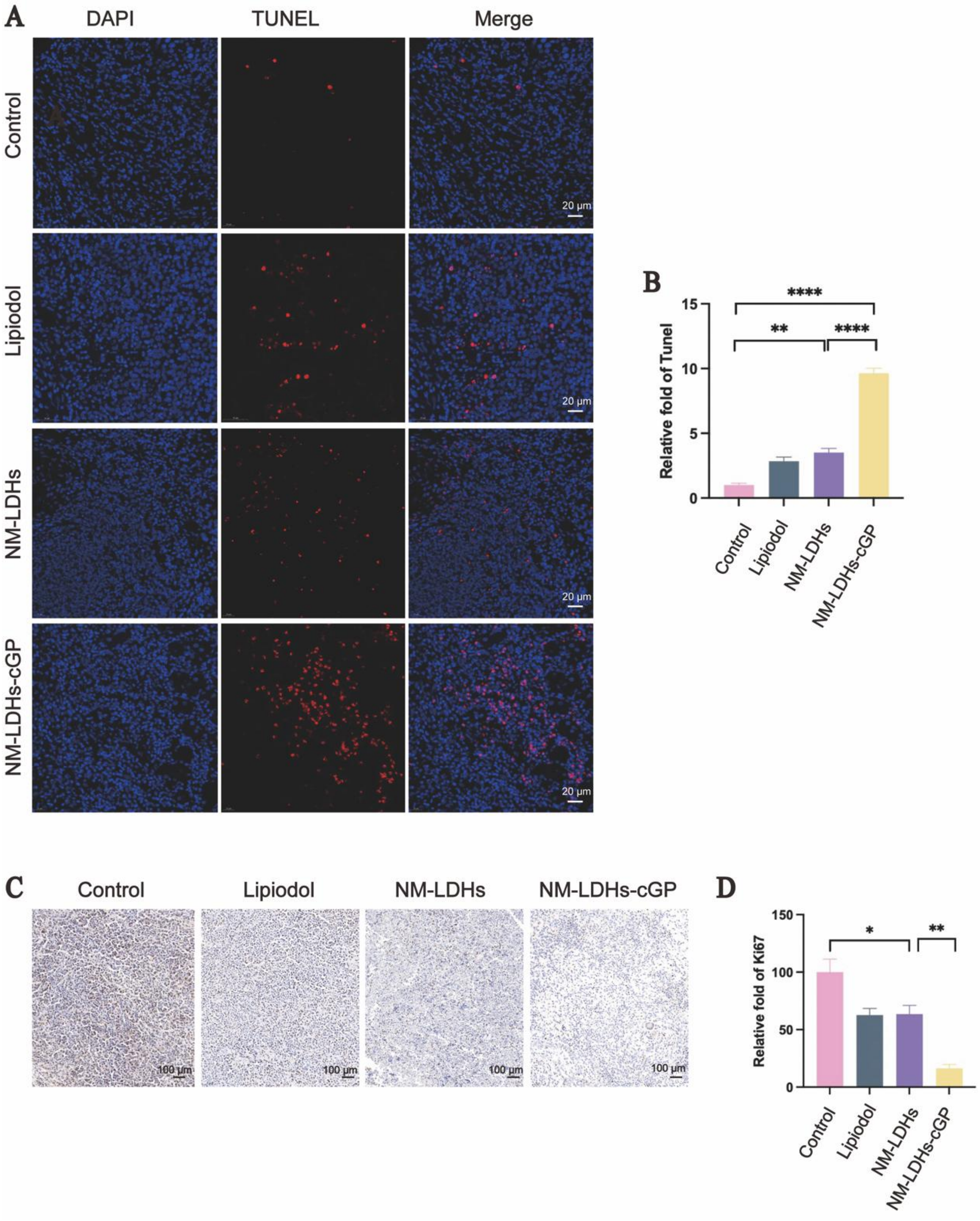

Supplementary Fig.9 A–B, TUNEL (A) and Ki67 (B) staining images along with their corresponding quantitative analysis graphs of tumor tissues after treatment with Lipiodol, NM-LDHs, and NM-LDHs-cGP. Scale bar: 20 μm (A) and 100 μm (C). Data presented as mean ± s.d. All replicates are biological. One-way ANOVA was performed for statistical significance. *$P < 0.05$, **$P < 0.01$, ***$P < 0.001$, ****$P < 0.0001$, ns, not statistical significance.

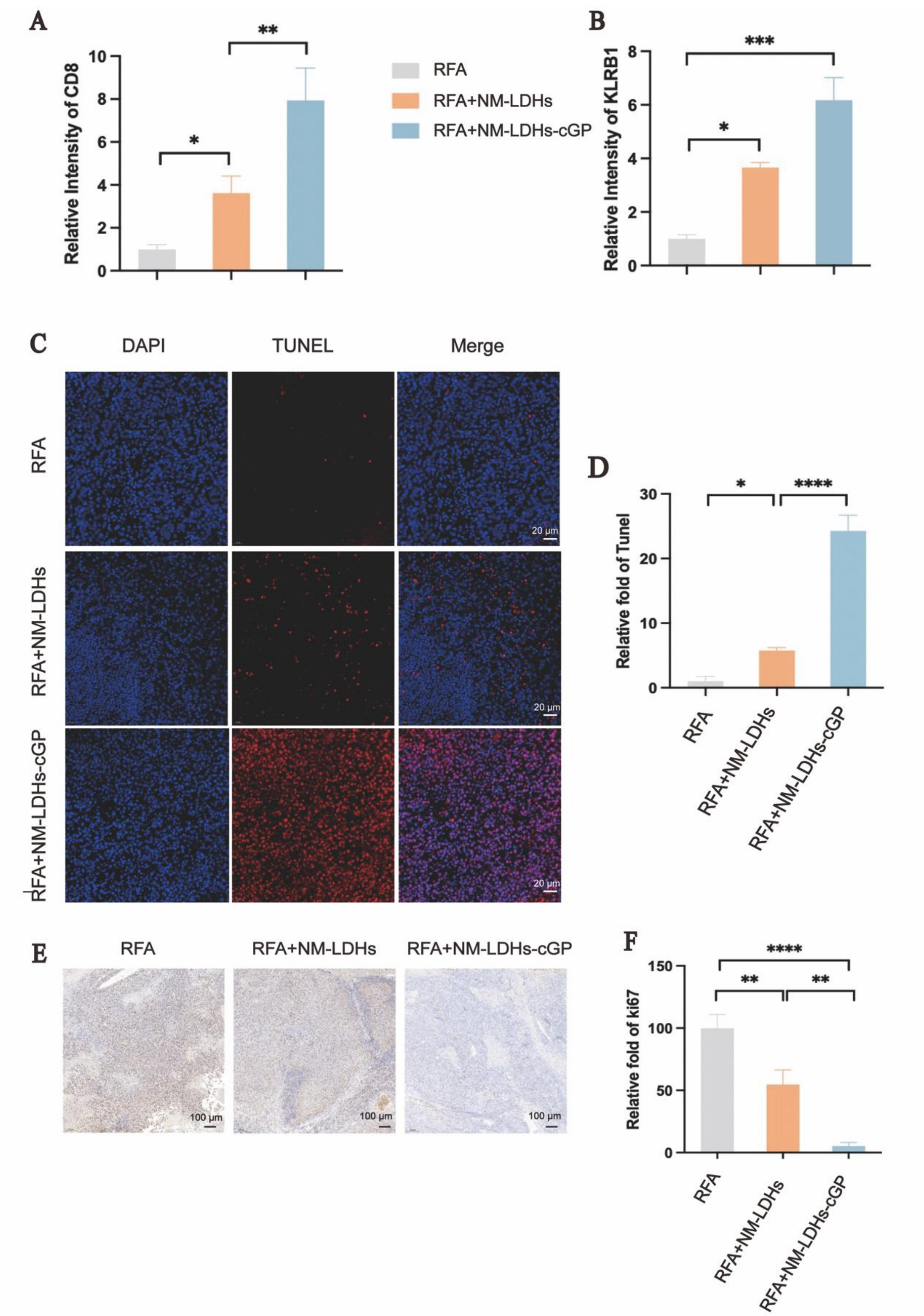

Supplementary Fig.10 A-B, Quantitative analysis graphs of CD8 (A) and KLRB1 (B) expression in tumor tissues after treatment with RFA, L-LDHs+RFA, L-LDHs-cGP+RFA. C-D, TUNEL staining images (C) along with their corresponding quantitative analysis graphs (D) of tumor tissues after treatment with RFA, L-LDHs+RFA, L-LDHs-cGP+RFA. Scale bar: 20 µm. E-F, Ki67 staining images

(E) along with their corresponding quantitative analysis graphs (F) of tumor tissues after treatment with RFA, L-LDHs+RFA, L-LDHs-cGP+RFA. Scale bar: 100 μm. Data presented as mean ± s.d. All replicates are biological. One-way ANOVA was performed for statistical significance. *$P < 0.05$, **$P < 0.01$, ***$P < 0.001$, ****$P < 0.0001$, ns, not statistical significance.

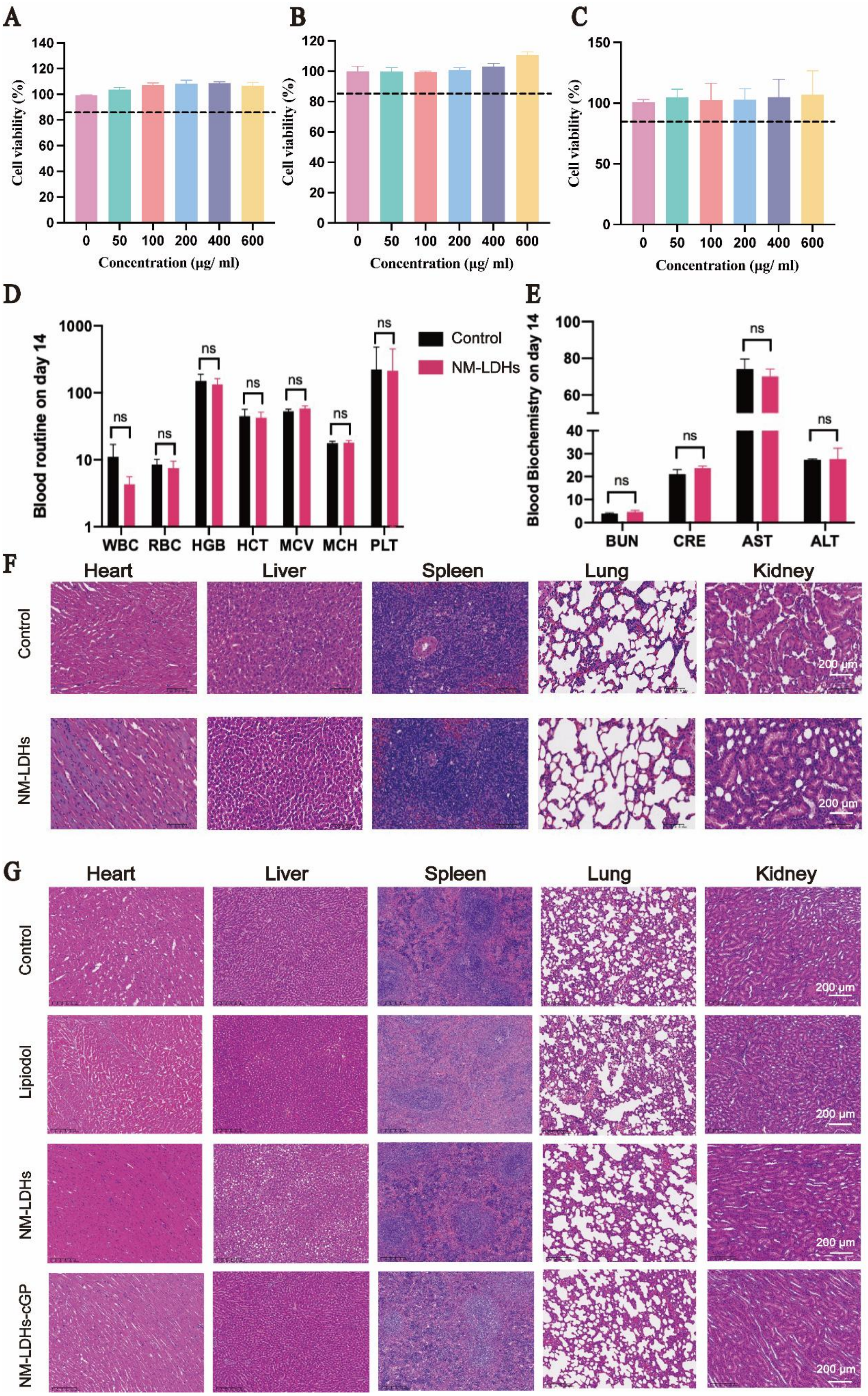

Supplementary Fig.11 A-C, Cell viability of Hepa1-6 (A), Huh-7 (B), and Thle-2 (C) after co-

incubation with different concentrations of NM-LDHs. D-E, Complete blood count (D) and blood biochemical (E) analysis results in rats from the single arterial embolization experiment at 14 days post-treatment. F-G, Histopathological changes in major organs (heart, liver, spleen, lung, and kidney) across different treatment groups. Scale bar: 200 μm. Data presented as mean ± s.d. All replicates are biological. Two-tailed Student's *t*-test was performed for statistical significance. *$P < 0.05$, **$P < 0.01$, ***$P < 0.001$, ****$P < 0.0001$, ns, not statistical significance.

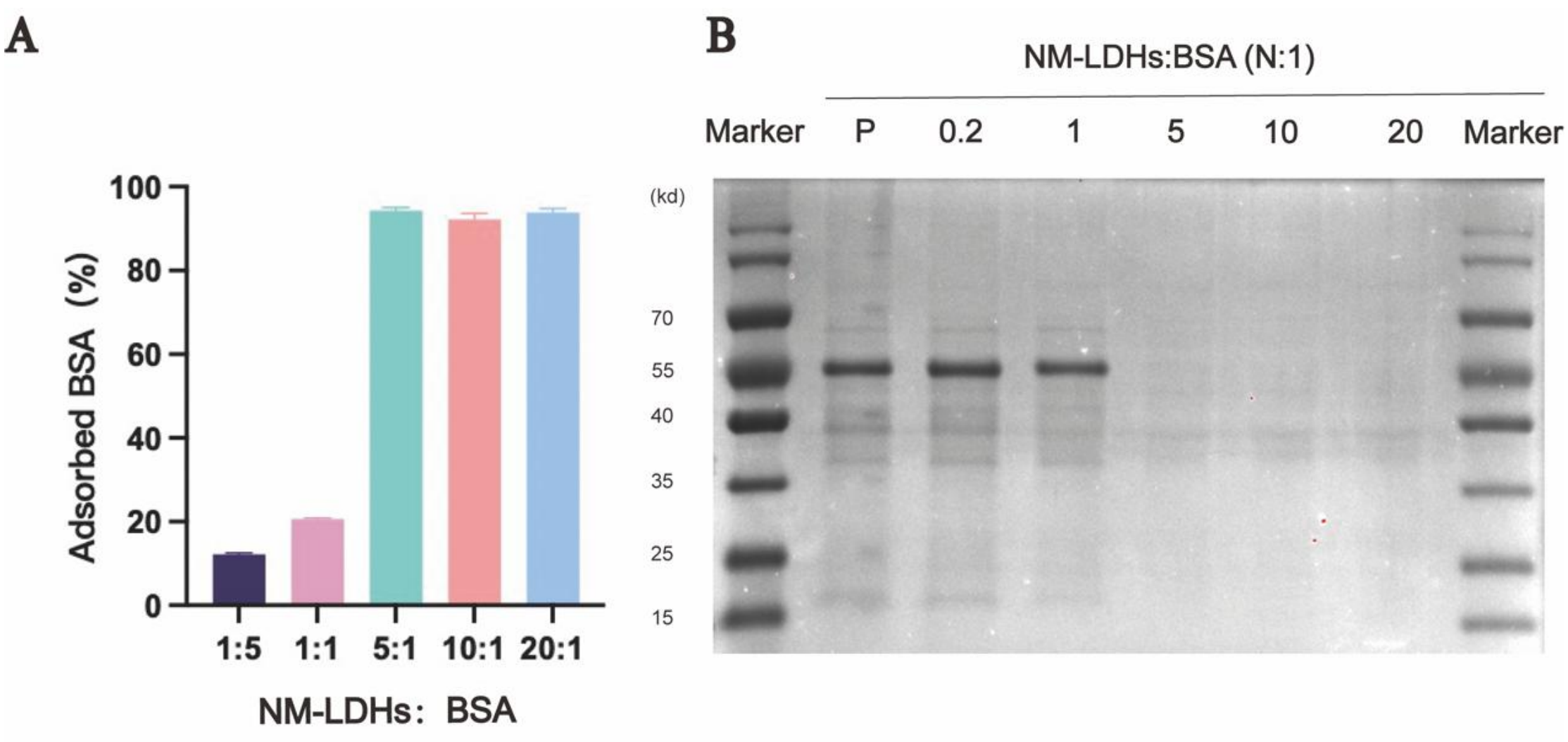

Supplementary Fig.12 A-B, Quantitative detection (A) by BCA assay and qualitative analysis (B) by SDS-PAGE of NM-LDHs adsorption with BSA at different mass ratios.

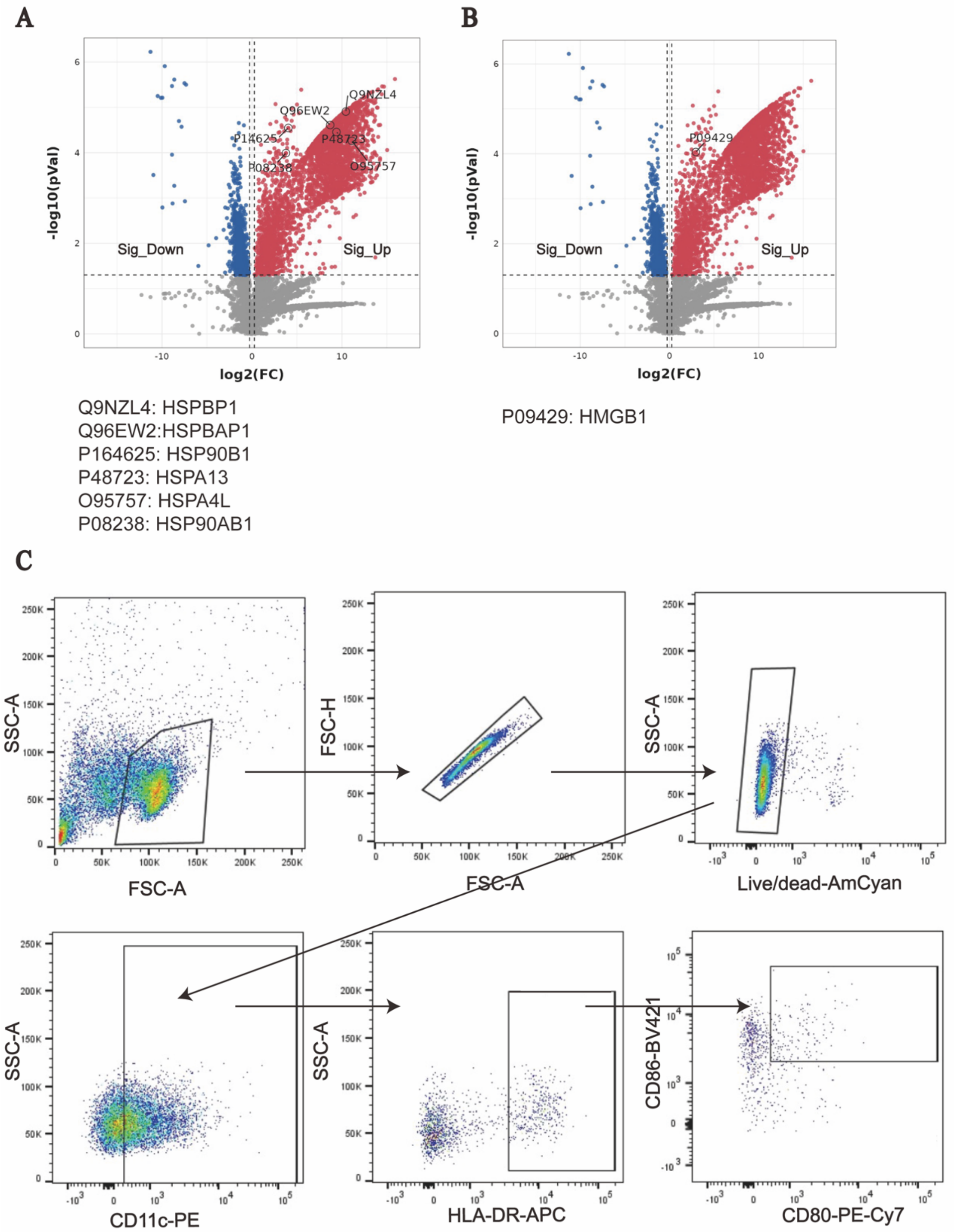

Supplementary Fig.13 A-B Volcano plot of differentially expressed proteins associated with HSP (A) and HMGB (B) in the supernatant before and after NM-LDHs-cGP adsorption (n = 3) . The horizontal axis represents log2(FC), and the vertical axis represents -log10(P-value). Red dots indicate upregulated proteins, and blue dots indicate downregulated proteins. C. Gating strategy for $CD80^+$ dendritic cells and $CD86^+$ dendritic cells.2

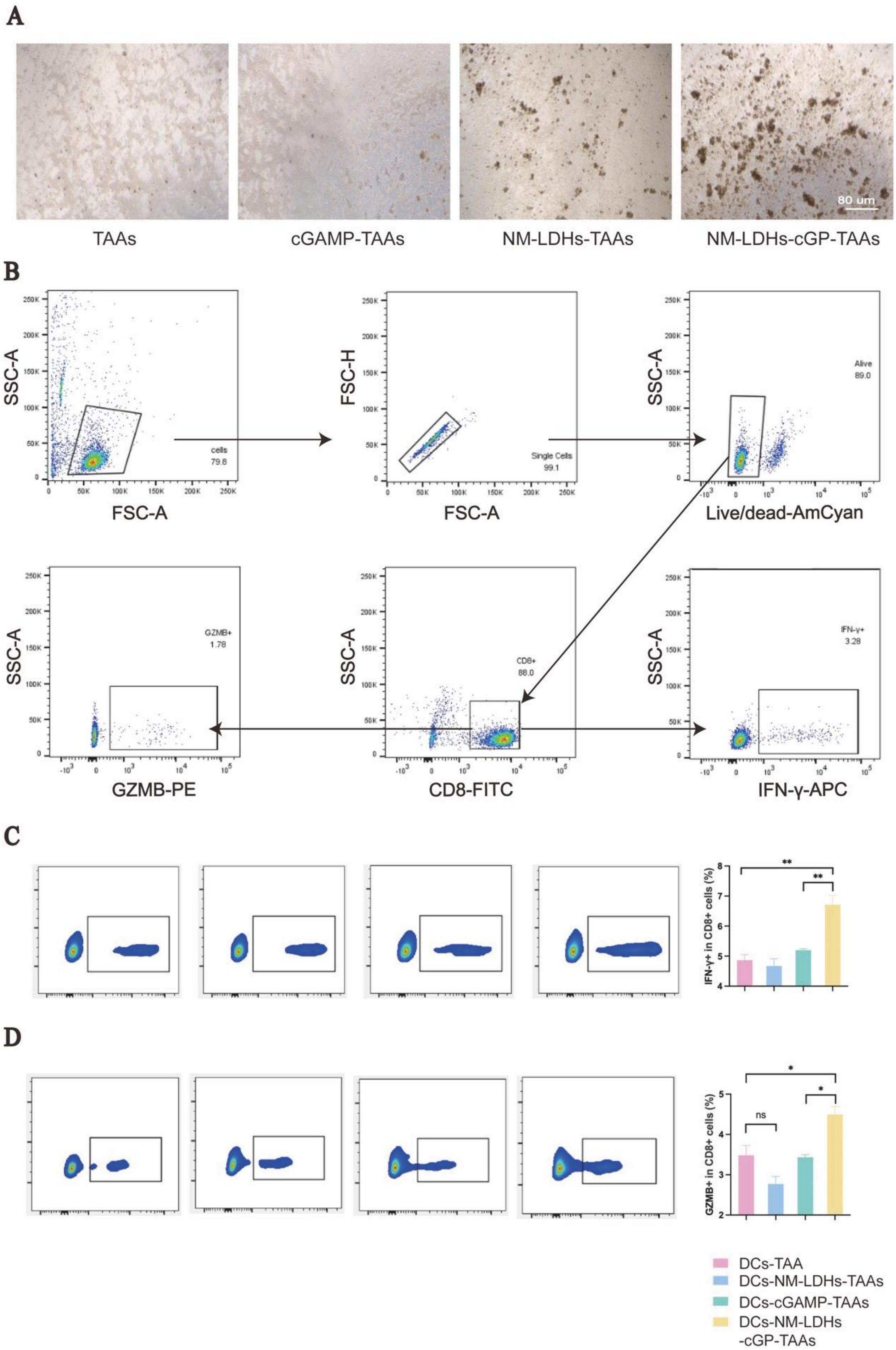

Supplementary Fig.14 A, Proliferation of T cells induced by different treatments. Scale bar: 80 μm. B, Gating strategy for IFN-γ+ CD8+ T cells and GZMB+ CD8+ T cells. C-D, Flow cytometry

analysis of IFN-γ+ C) and GZMB (D) expression in human T cells co-cultured with differently treated human dendritic cells for 7 days (n = 3). Data presented as mean ± s.d. All replicates are biological. One-way ANOVA was performed for statistical significance. *$P < 0.05$, **$P < 0.01$, ***$P < 0.001$, ****$P < 0.0001$, ns, not statistical significance.

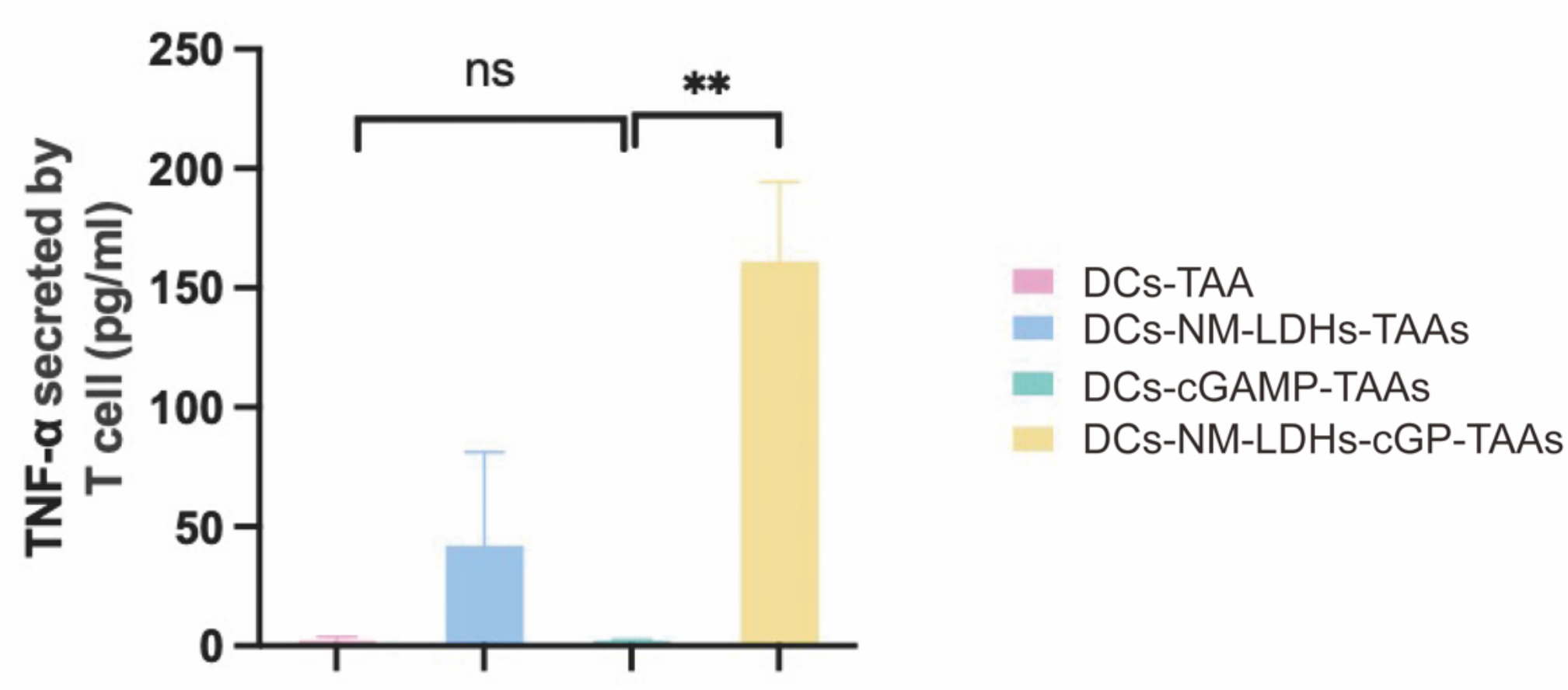

Supplementary Fig.15 A, Secretion levels of TNF-α in the culture supernatant after co-culturing human T cells with differently treated human dendritic cells for 10 days (n = 3). Data presented as mean ± s.d. All replicates are biological. One-way ANOVA was performed for statistical significance. *$P < 0.05$, **$P < 0.01$, ***$P < 0.001$, ****$P < 0.0001$, ns, not statistical significance.
